# A genome-wide function of THSC/TREX-2 at active genes prevents transcription–replication collisions

**DOI:** 10.1093/nar/gku906

**Published:** 2014-10-07

**Authors:** José M. Santos-Pereira, María L. García-Rubio, Cristina González-Aguilera, Rosa Luna, Andrés Aguilera

**Affiliations:** Centro Andaluz de Biología Molecular y Medicina Regenerativa CABIMER, Universidad de Sevilla-CSIC, 41092 Seville, Spain

## Abstract

The THSC/TREX-2 complex of *Saccharomyces cerevisiae* mediates the anchoring of transcribed genes to the nuclear pore, linking transcription elongation with mRNA export and genome stability, as shown for specific reporters. However, it is still unknown whether the function of TREX-2 is global and the reason for its relevant role in genome integrity. Here, by studying two TREX-2 representative subunits, Thp1 and Sac3, we show that TREX-2 has a genome-wide role in gene expression. Both proteins show similar distributions along the genome, with a gradient disposition at active genes that increases towards the 3′ end. Thp1 and Sac3 have a relevant impact on the expression of long, G+C-rich and highly transcribed genes. Interestingly, replication impairment detected by the genome-wide accumulation of the replicative Rrm3 helicase is increased preferentially at highly expressed genes in the *thp1Δ* and *sac3Δ* mutants analyzed. Therefore, our work provides evidence of a function of TREX-2 at the genome-wide level and suggests a role for TREX-2 in preventing transcription–replication conflicts, as a source of genome instability derived from a defective messenger ribonucleoprotein particle (mRNP) biogenesis.

## INTRODUCTION

During transcription from RNA polymerase II (RNAPII), the nascent pre-mRNA is bound by RNA-binding proteins and undergoes a series of processing steps, including 5′-end capping, splicing and 3′-end cleavage and polyadenylation (1). This leads to the formation of the messenger ribonucleoprotein particle (mRNP), which is co-transcriptionally exported to the cytoplasm through the nuclear pore complex (NPC) (2). A variety of factors participate in mRNP export in *Saccharomyces cerevisiae*, including the conserved THO complex, the heterogeneous nuclear ribonucleoproteins (hnRNPs) Nab2 and Npl3, the export receptor Mex67-Mtr2 and the NPC-associated THSC/TREX-2 complex. The current view is that these factors act in a sequential process that guides the mRNA to the NPC to be exported to the cytoplasm (3). In this sense, increasing evidences suggest that highly transcribed genes and some inducible genes are recruited to the nuclear envelope upon transcriptional activation in yeast and higher eukaryotes (4). In agreement with the gene gating hypothesis (5), the positioning of transcribed genes to the proximity of the NPC is dependent on a variety of factors, including those involved in transcription initiation and mRNA export (6–11).

The yeast THSC/TREX-2 complex (TREX-2 from now on), composed by Sac3, Thp1, Sus1, Cdc31 and Sem1, is an important mediator of mRNA export that provides an anchoring of transcribed genes to the NPC (12–17). Sac3, the largest subunit of the complex, acts as a platform with different interaction domains. Whereas its N-terminus associates with Thp1 and Sem1 to form an mRNA-binding module, as well as with the export receptor Mex67, its C-terminus interacts with Sus1, Cdc31 and the Nup1 nucleoporin, providing anchoring to the NPC (13,18–21). This organization is highly conserved from yeast to human cells (18,19,22,23). In addition to TREX-2, Sus1 is also a component of the histone H2B deubiquitinylation module of the SAGA complex, involved in transcription initiation of some yeast genes (15,24). It has been shown that Thp1, Sac3 and Sus1 are required for proper relocation of active genes to the NPC upon transcriptional activation (6,8,10). Cdc31 is the yeast homolog of the human centrins, functions in the duplication of the spindle pole body and is important for mRNA export (14,25). Finally, Sem1 is a common component of the 19S regulatory particle of the 26S proteasome, the COP9 signalosome and TREX-2, and is also important for transcription initiation and mRNA export (16,17).

In addition to the export of mRNAs, the yeast TREX-2 complex plays a role in transcription elongation, as has been shown using artificial reporters such as the G+C-rich gene *lacZ* or a G-less-based run-on (GLRO) assay (12,26,27). This function is common to other mRNA export factors such as the THO complex (28), and THO mutants show a genome-wide down-regulation of long, G+C-rich and highly expressed genes (29). Interestingly, recent works have demonstrated that several mRNA export factors associate with the chromatin of transcribed genes all over the genome (29–31), suggesting a general role of these proteins in transcription. However, no evidence exists for a genome-wide function of TREX-2 in transcription, and whether or not its components bind to genes remains unknown.

An important feature of yeast TREX-2 mutants is their high level of transcription-associated hyperrecombination (TAR), which supports a connection among mRNA export, transcription and genome instability (12,16,26,32). The TAR phenotype is common to other mutants involved in transcription and mRNA export, such as THO, Sub2, Npl3 or Mex67, as well as other RNA processing mutants from yeast to human cells (30,33,34). TAR is believed to occur as a consequence of several mechanisms. First, a putatively stalled RNAPII would increase negative supercoiling behind transcription, leading to the accumulation of single-stranded DNA (ssDNA). This ssDNA is more susceptible to the action of nucleases, genotoxic agents and the human activation-induced cytidine deaminase (AID), as has been shown for TREX-2 and other related mutants (26,30,35). Second, transcription would constitute an obstacle for replication fork (RF) progression, leading to strong replication impairment at transcribed genes (29). However, there is no evidence connecting TAR and replication in TREX-2 mutants.

In this work, the genome-wide function of TREX-2 is analyzed. Thp1 and Sac3 mutations are shown by microarray analyses to lead to a down-regulation of long, G+C-rich and highly transcribed genes. Comparison of TREX-2 mutant expression profiles with those of THO mutants supports the idea that Thp1 and Sac3 act as a functional unit, playing a role in gene expression different from that of the THO complex. By ChIP-chip, we show that Thp1 and Sac3 bind to highly transcribed genes in a gradient manner towards their 3′ ends, similar to the THO subunit Hpr1. Interestingly, depletion of Thp1 or Sac3 leads to a strong genome-wide replication impairment detected by accumulation of the replicative Rrm3 helicase at highly transcribed genes and other regions that is stronger in *sac3Δ* cells. Taken together, our results provide evidence that Thp1 and Sac3 function in highly expressed genes all over the genome and help prevent transcription–replication collisions that cause genome instability.

## MATERIALS AND METHODS

### Yeast strains and molecular biology

Plasmids pCM184 and pCM184RNH1 were described previously (30,36). Yeast strains used in this study are listed in Supplementary Table S2. Yeast genetics and molecular biology methodologies, including protein extractions and westerns, were performed using standard procedures.

### Microarray gene expression analysis

Microarray determination of total RNA was performed using the Affymetrix platform, as previously described (29). Briefly, total RNA was isolated from wild-type and mutant strains and the RNA was extracted using the RNeasy Midi Kit (Qiagen). RNA levels for all yeast genes were determined using an Affymetrix microarray scanner. For each strain, microarray analysis was conducted in triplicate and the values presented represent the average of these three determinations. The expression data can be accessed at Gene Expression Omnibus (GSE56703, GSE56702).

### Microarray validation by RT-qPCR

Total RNA was extracted from exponentially growing cells using the RNeasy Mini Kit (Qiagen) and cDNA was obtained by the Quantitec Reverse Transcription Kit (Qiagen). qPCR reactions were performed from 1 μl of cDNA following the manufacturer's instructions. RNA levels were determined by relative quantitation normalized to the *SCR1* gene, transcribed by RNAPIII. Primers used are listed in Supplementary Table S3.

### ChIP-chip experiments

*S. cerevisiae* oligonucleotide tiling microarrays were provided by Affymetrix. The high-density oligonucleotide arrays used are able to analyze yeast chromosomes at a 300-bp resolution, each of the 300-bp region being covered by at least 60 probes. ChIP-chip of asynchronously growing cells was carried out as described (37,38). Briefly, we disrupted 1.5×10^7^ cells by multi-beads shocker (MB400U, Yasui Kikai, Japan), which was able to keep cells precisely at lower than 6°C during disruption by glass beads. Anti-FLAG monoclonal antibody M2 (Sigma-Aldrich) was used for ChIP. ChIP DNA was purified and amplified by random priming using a WGA2 kit (Sigma-Aldrich) and following the manufacturer's procedure. A total of 4 μg of amplified DNA was digested with DNase I to a mean size of 100 bp, purified, and the fragments were end-labeled with biotin-N6-ddATP23. ChIP-chip experiments shown with Thp1-FLAG and Sac3-FLAG are one per strain analyzed, which gave identical results. Rrm3-FLAG results shown are from two repetitions made for each strain analyzed, the data being normalized by the quantile method. The ChIP-chip data can be accessed at Gene Expression Omnibus (GSE56703, GSE56700).

### Statistical analysis of genome-wide data

Microarray data analysis of gene expression was performed using the dChip application software (http://www.dchip.org) (39). All arrays were normalized with PM probes (invariant set normalization and model-based expression) and the outliers treated as missing data in the subsequent analyses. A filter was used to select only *S. cerevisiae* genes. Model-based expression values are represented as arbitrary units (A.U.) in linear scale. For each strain, the expression profile was compared with its isogenic wild type and the genes showing at least a 1.5-fold expression change considered as altered (parameters: absolute difference between signal in mutant versus wild type >100; difference *P*-value <0.05). Genes with model-based expression values below 600 (the median expression value of meiotic genes in our experiments) in at least 50% of the samples were removed before the comparisons to reduce the false positives. Heatmaps were done with the Multiexperiment Viewer application software (http://www.tm4.org) considering the 50% of genes with major signal fold-change. Hierarchical clustering was performed by the complete linkage method according to a Pearson's correlation.

ChIP-chip data were analyzed using the Tiling Array Suite software (TAS) from Affymetrix, as previously described (30). TAS produces, per probe position, the signal and the change *P-*value, taking into account the probes localized within a given bandwidth around the inspected probe. Protein chromosomal distribution was then analyzed by detecting binding clusters, which were defined as ranges within the chromosome respecting the following conditions: estimated signal (IP/SUP-binding ratio) positive in the whole range; *P*-value<0.01, minimum run of 100 bp and maximum gap of 250 bp. The results were visualized with the UCSC Genome Browser, developed and maintained by the Genome Bioinformatics Group (Center for Biomolecular Science and Engineering at the University of California, Santa Cruz) (http://genome.ucsc.edu/). For statistical analysis of the functional and structural features of the genes, model-based expression levels were taken from microarray of W303-1A (29). Distribution of binding sites along genes was carried out as previously described (29).

### ChIP analyses

Recruitment of Rrm3 to chromatin was determined by ChIP analyses as previously described (40). The Wizard SV Gel and PCR clean-up system (Promega) was used for the last DNA purification step. Primers used are listed in Supplementary Table S3. The PCR product of the intergenic region at positions 9716–9863 of chromosome V was used as a negative control. Quantitative PCR and calculations of the relative abundance of each DNA fragment were performed as described (41). For each experiment, the DNA ratios in the different regions were calculated relative to the amount of the intergenic region. Average and standard error of the mean of three independent experiments are shown.

## RESULTS

### Thp1 and Sac3 act as a functional unit in the regulation of global gene expression

Transcription from artificial reporters is impaired in TREX-2 mutants, a phenomenon linked to their mRNA export defects and TAR phenotype (12,26,27). To assess whether these transcription defects apply to the whole genome, microarray analyses of gene expression were performed in *thp1Δ*, *sac3Δ* and *sus1Δ* cells, comparing the data to those of the THO mutants *hpr1Δ*, *tho2Δ* and *sub2Δ* (29). Genes with mRNA levels at least 1.5-fold above or below the wild-type levels and a *P* < 0.05 were selected for analysis. We excluded those with expression values below the median for meiotic genes in our experiments, taken as the cutoff for non-expression values, in order to reduce the false positives. We found a different number of de-regulated genes in the six mutant strains, *tho2Δ*, *sub2Δ* and *thp1Δ*, these being the mutants with the highest impact on gene expression, with 711, 912 and 992 de-regulated genes, respectively (Supplementary Table S1).

**Figure 1.**
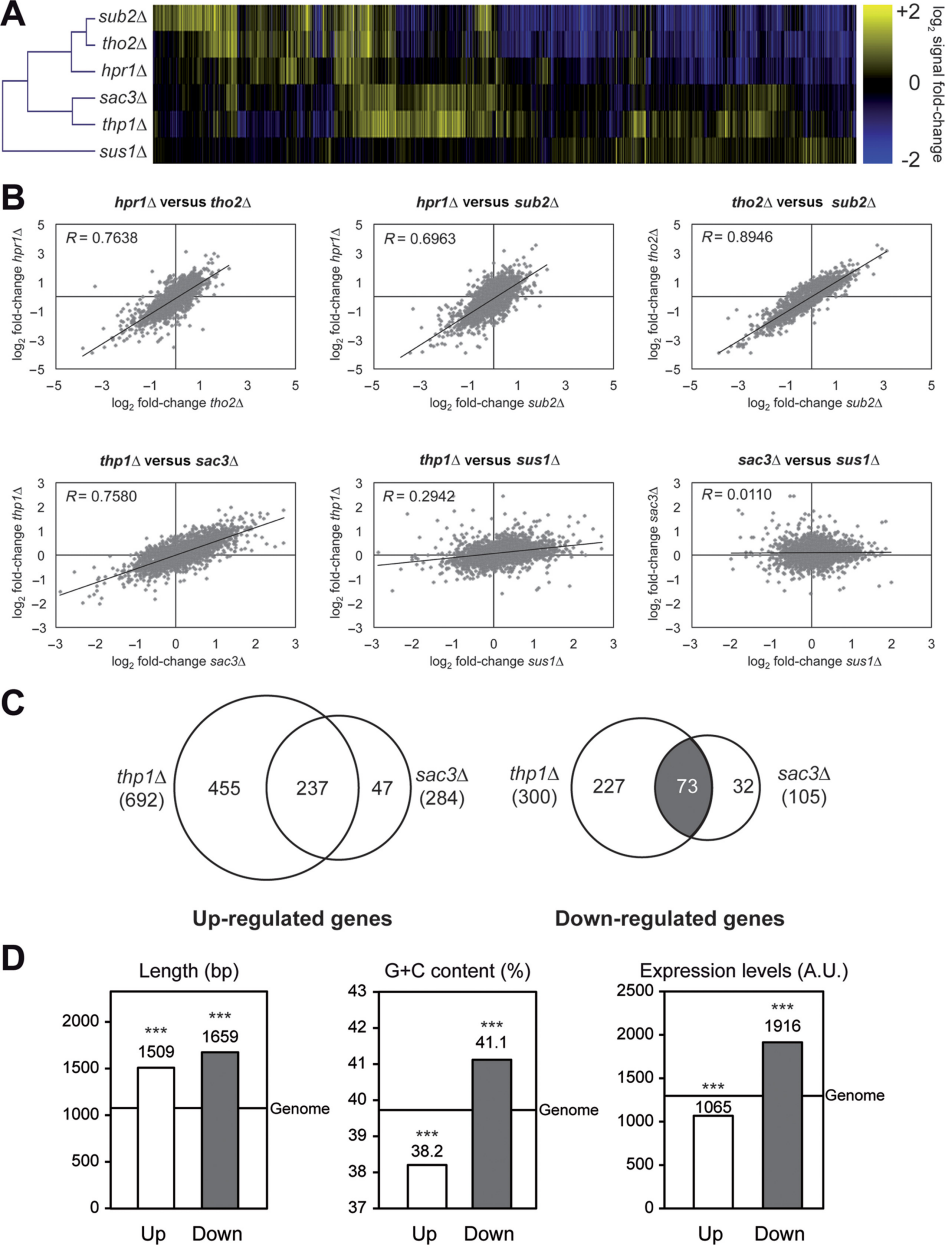
Microarray expression analysis of genes affected by THO, Sub2 and THSC/TREX-2 mutations. (**A**) Clustering of the expression profiles of THO–Sub2 and Thp1–Sac3 mutants. Heat-map of the 50% of genes with major log_2_ signal fold-change in THO, Sub2 and THSC/TREX-2 mutants compared to wild type is shown. (**B**) Correlation analysis between the expression profiles of THO–Sub2 and THSC/TREX-2 mutants. Scatter plots showing correlation among the log_2_ signal fold-change of THO–Sub2 (top) and THSC/TREX-2 (bottom) mutants. A linear regression line and the Pearson's R correlation coefficient are plotted per graph. (**C**) Overlap between de-regulated genes in *thp1Δ* and *sac3Δ* mutants. Venn diagrams representing the genes whose expression is significantly changed (*P* < 0.05, fold-change > 1.5) in *thp1Δ* and *sac3Δ* cells. The complete list of genes can be found in the Supplementary Dataset II. (**D**) Functional and structural features of the de-regulated genes in *thp1Δ* and *sac3Δ* mutants. Statistical analysis of length, G+C content and model-based expression levels for genes whose expression levels are coincidently affected in *thp1Δ* and *sac3Δ* cells. Median values are shown and a line represents the genome median. ***, *P* < 0.001 (Mann–Whitney's *U*-test) as compared to the genome median. Genes showing expression levels below the median of the meiotic genes in our experiments in at least 50% of the samples were removed from the comparison.

Hierarchical clustering of the mutant expression profiles showed that *thp1Δ* and *sac3Δ* were clearly similar between them but different from THO and *sub2Δ* mutants (Figure 1A). This is consistent with Thp1 and Sac3 being a structural and functional unit working in mRNA export (12,18). *sus1Δ* cells had the most different profile from the other mutants. Even though small differences could be observed between *thp1Δ* and *sac3Δ* expression profiles (Figure 1A), a high correlation was seen between the mutant expression profiles of both mutants (Pearson's *R* correlation coefficient = 0.7580), as well as between those of THO mutants and *sub2Δ* (*R* = 0.6963 – 0.8946) (Figure 1B). However, the profiles of these five mutants did not correlate with that of *sus1Δ* (*R* = –0.1974 – 0.2942; Figure 1B and Supplementary Figure S1A). We conclude that Thp1 and Sac3 work as a functional unit modulating gene expression, the effects of their depletion being different from those of THO, Sub2 and Sus1. Therefore, we decided to continue the work with Thp1 and Sac3, as the most functionally significant subunits.

We then analyzed the genes whose expression was de-regulated in *thp1Δ* and *sac3Δ* cells according to the microarray data. Consistent with the clustering results, we observed a high overlap between up- and down-regulated genes in both strains (*P* < 0.0001), with most up- (83%) and down-regulated genes (70%) in *sac3Δ* cells being coincident with those of *thp1Δ* (Figure 1C). This overlap was lower when compared with de-regulated genes of *hpr1Δ* cells, as only 18–36% of de-regulated genes in *hpr1Δ* overlapped with those of *thp1Δ* or *sac3Δ* (Supplementary Figure S1B). Statistical analysis of the structural and functional features of the 237 up-regulated and 73 down-regulated genes that were coincident between *thp1Δ* and *sac3Δ* cells showed that both groups of genes were longer than the genome median (Figure 1D; *P* < 0.001). Whereas up-regulated genes were G+C-poor and poorly expressed, down-regulated genes were G+C-rich and highly expressed (Figure 1D; *P* < 0.001), similar to the results of THO mutants (Supplementary Figure S2), and in contrast to the general tendency of long genes in yeast that are G+C poor (42). Gene ontology analyses showed a high variety of biological processes enriched in the up-regulated genes, such as transcription, mRNA processing, chromosome segregation and cell cycle (Supplementary Dataset I), while down-regulated genes were only enriched in ion transport genes, suggesting that this down-regulation was specific of long, G+C-rich and highly expressed genes, independent of function. Changes in gene expression were validated by RT-qPCR for some up- and down-regulated genes in *thp1Δ* and *sac3Δ* cells as indicated (Supplementary Figure S3). Altogether, our results indicate that Thp1 and Sac3 have a relevant function in the expression of highly transcribed genes.

### Genome-wide recruitment of Thp1 and Sac3 to active genes

**Figure 2.**
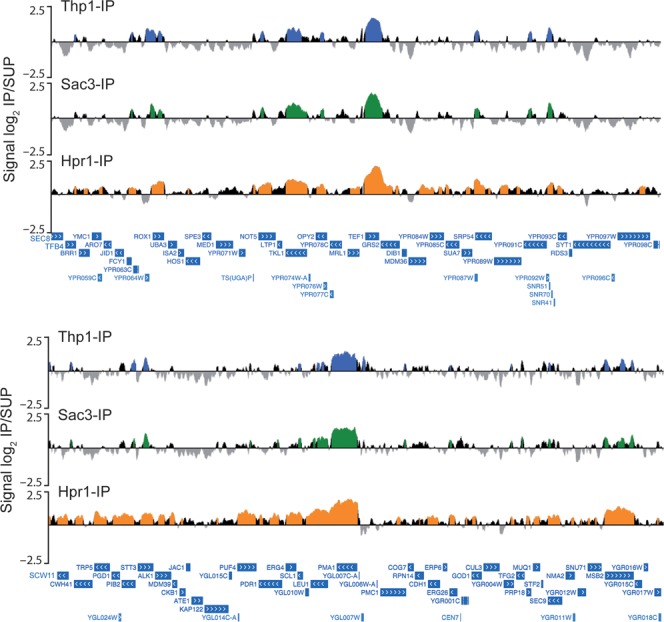
Recruitment pattern of Thp1, Sac3 and the THO complex to ORFs. ChIP-chip analysis of Thp1-FLAG, Sac3-FLAG and Hpr1-FLAG binding to the whole genome. Two genomic fragments from chromosomes XVI and VII are shown, with enrichment values represented as signal log_2_ IP/SUP ratio. Blue (Thp1-IP), green (Sac3-IP) and orange (Hpr1-IP) histograms (online version) represent the significant binding clusters (*P* < 0.01, minimum run >100 bp, maximum gap <250 bp). Genes and other features are represented according to the *Saccharomyces* Genome Database (SGD) as blue bars and white arrows in relation to the direction of transcription.

**Figure 3.**
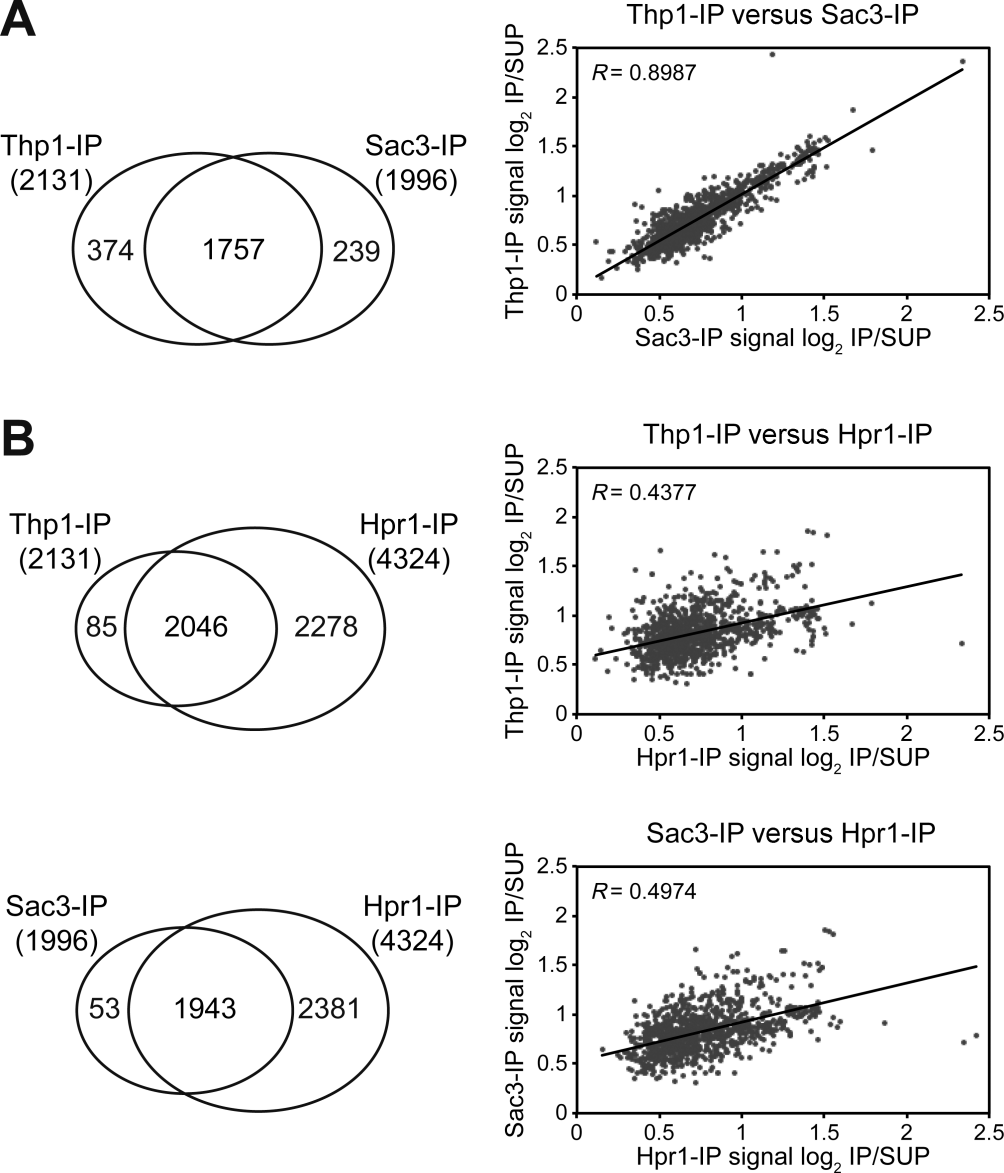
Analysis of Thp1- and Sac3-enriched genes compared to Hpr1. (**A**) Correlation between Thp1- and Sac3-enriched genes. Left, Venn diagram showing the overlap between Thp1- and Sac3-enriched genes in ChIP-chip analyses. Right, scatter plot showing correlation between Thp1 and Sac3 enrichment levels in the common ORFs. (**B**) Correlation among Thp1-, Sac3- and Hpr1-enriched genes. Left, Venn diagrams showing the overlap between Thp1-, Sac3- and Hpr1-enriched genes in ChIP-chip analyses. Right, scatter plots showing correlation between Thp1, Sac3 and Hpr1 enrichment levels in the common ORFs. A linear regression line and the Pearson's R correlation coefficient are plotted per scatter plot. The complete list of genes can be found in the Supplementary Dataset II.

To analyze the binding of Thp1 and Sac3 to the genome, ChIP-chip experiments were performed with yeast strains carrying both proteins FLAG-tagged. ChIP-chip data were subjected to computational analysis to obtain a genomic map distribution. We found that binding of the two TREX-2 subunits to the genome was highly similar (*R* = 0.9192; Figure 2). Statistical analysis revealed that 69.3% and 69.8% of Thp1 and Sac3 binding clusters, respectively, peaked inside Open Reading Frames (ORFs). Thp1 and Sac3 were significantly enriched in a similar number of genes (2131 and 1996, respectively), most of them coincident (74.1%; *P* < 0.0001) and with a high correlation of recruitment levels (*R* = 0.8987; Figure 3A). These results indicate that Thp1 and Sac3 act as a complex that associates with transcribed DNA regions.

We then compared the distribution of Thp1 and Sac3 with that of the THO subunit Hpr1, previously published (29), and observed that genes associated with Thp1 and Sac3 were also enriched in Hpr1 (Figures 2 and 3B). Correlations between Hpr1 and Thp1 or Sac3 enrichment levels were significant but lower than those of Thp1 and Sac3 (*R* = 0.4377 and 0.4974, respectively; *P* < 0.001; Figure 3B). We conclude that Thp1 and Sac3 function in the genes to which THO also binds. Additionally, Thp1 and Sac3 were also found at telomeres and some ARSs, introns, snRNAs, snoRNAs, and RNAPIII-transcribed genes, as is also the case of Hpr1 (Table 1). Sac3 was also found at seven centromeres, where non-significant peaks of Thp1 were also detected (Supplementary Figure S4). This may suggest an additional function of Sac3 (and maybe Thp1) in chromosome segregation. Altogether, these data suggest a role of Thp1 and Sac3 in transcribed genes all over the genome, most of which share a similar pattern of THO distribution.

**Table 1.** Genomic features mapped by different ChIP-chip binding clusters

Genomic feature	Thp1-IP	Sac3-IP	Hpr1-IP	Rrm3-IP WT	Rrm3-IP *thp1Δ*	Rrm3-IP *sac3*Δ	Total genome
*ORFs*	2131	1996	4324	3926	4190	5520	6607
*Centromeres*	0	7	0	4	12	16	16
*Telomeres*	30	30	30	30	30	31	32
*ARS*	42	45	78	89	87	222	337
*Introns*	74	105	136	121	191	245	376
*snRNAs and snoRNAs*	15	23	35	23	61	68	83
*RNAPIII-transcribed*	36	39	57	75	211	237	309

### Preferential accumulation of Thp1 and Sac3 at the 3′ end of highly transcribed genes

**Figure 4.**
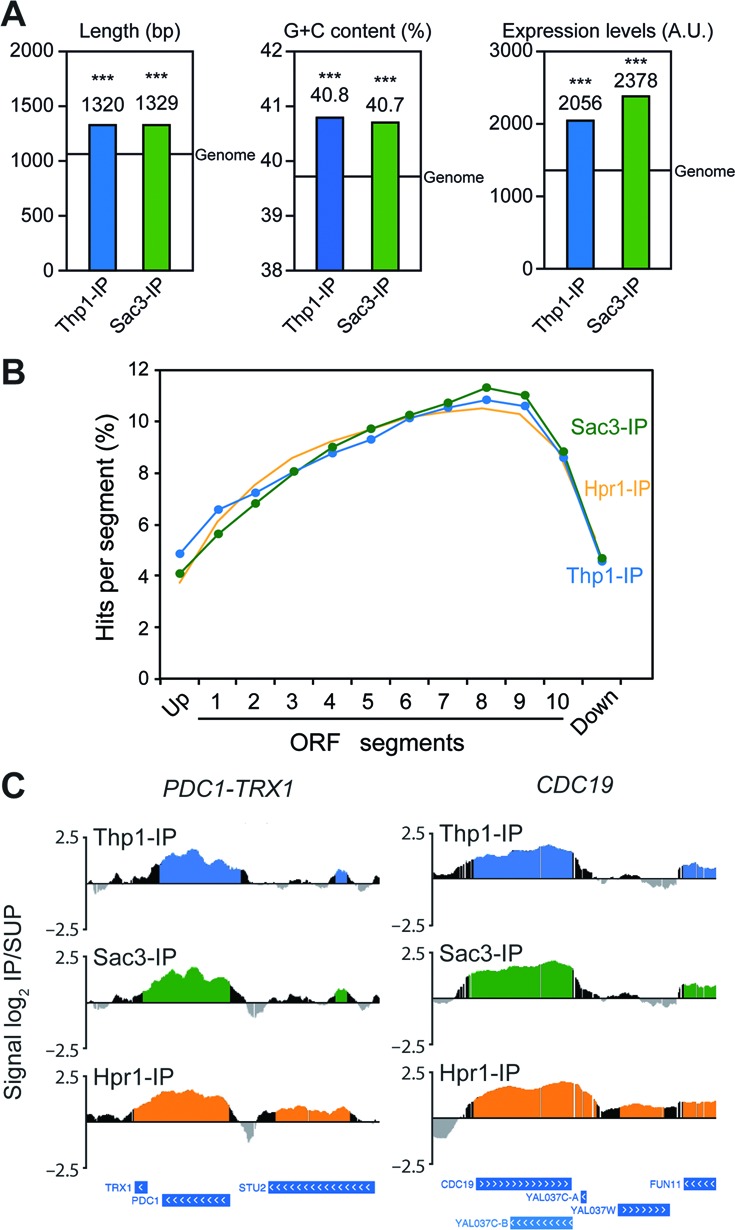
Thp1 and Sac3 accumulate towards the 3′ end of highly transcribed genes. (**A**) Functional and structural features of Thp1- and Sac3-enriched genes. Statistical analysis of length, G+C content and model-based expression levels for genes mapped by Thp1 and Sac3 clusters obtained in ChIP-chip experiments. Median values are shown; a line represents the genome median. ***, *P* < 0.001 (Mann–Whitney's *U*-test) as compared to genome median. Genes showing expression levels below the median of the meiotic genes in our experiments in at least 50% of the samples were removed from the comparison. (**B**) Distribution of Thp1 and Sac3 along the genes. Composite profile of Thp1 and Sac3 occupancy detected by ChIP-chip across the average ORF plotted as Thp1 or Sac3 percentage of ChIP clusters per segment. For this analysis, ORFs were subdivided in 10 segments independently of the ORF size plus two additional upstream and downstream segments of the same length. The binding distribution of Hpr1 (29) is shown for comparison. (**C**) Binding of Thp1 and Sac3 to highly expressed genes in an increasing gradient towards their 3′ end. Thp1, Sac3 and Hpr1 distribution at the highly transcribed *PDC1-TRX1* and *CDC19* loci, represented as signal log_2_ IP/SUP ratio. Blue (Thp1-IP), green (Sac3-IP) and orange (Hpr1-IP) histograms (online version) represent the statistically significant binding clusters (*P* < 0.01, minimum run >100 bp, maximum gap <250 bp). Genes and other features are represented according to the SGD.

Next, we analyzed the structural and functional features of the TREX-2-bound genes detected in the ChIP-chip experiments. We found that they were significantly longer, G+C-richer and more highly expressed than the genome median (*P* < 0.001; Figure 4A). Gene ontology analyses of these ORFs revealed that they were enriched in a large variety of biological processes (Supplementary Dataset I), specially ribosome-related genes, which are highly expressed genes in yeast. Interestingly, a good correlation was found between Thp1 and Sac3 binding and expression levels in TREX-2-enriched genes, similar to that obtained for Hpr1 (Supplementary Figure S5). This suggests that the higher the expression level of the gene, the higher the detection capacity of these proteins by ChIP. In addition, we found that Thp1-bound genes significantly overlapped with down-regulated genes in *thp1Δ* cells (*P* < 0.05), but not with up-regulated genes (*P* = 0.2926; Supplementary Figure S6), in agreement with a specific down-regulation of the genes to which Thp1 binds. This effect was not clearly seen in Sac3-bound genes, probably due to the lower number of de-regulated genes in *sac3Δ* cells (Supplementary Figure S6). We conclude that TREX-2 accumulates at highly transcribed chromatin, consistent with its role in transcription and mRNA export.

Finally, the relative distribution of Thp1 and Sac3 along the length of the ORFs was analyzed by subdividing them into 10 equal segments independent of ORF size. Additional upstream (5′) and downstream (3′) segments of the same length were taken as promoter and terminator regions, respectively, as previously described (29). The distribution of the proteins along the ORFs was determined by the percentage of ChIP-chip clusters mapping on each segment. We found that Thp1 and Sac3 distribution increased gradually towards the 3′ end of the genes, while they bind poorly to the promoter and terminator regions, similarly to Hpr1 (Figure 4B and C). To assess whether Thp1 and Sac3 loading to the genes was dependent on the nascent mRNA, we analyzed their distribution along genes of different length intervals. As shown in Supplementary Figure S7, this distribution was independent of gene length, as the gradient recruitment of TREX-2 proteins to the 3′ end occurred in a similar manner in gene populations of different lengths. Altogether, these results suggest that Thp1 and Sac3 bind co-transcriptionally to highly expressed genes, accumulating preferentially at their 3′ ends.

### Genome-wide replication impairment in *sac3Δ* cells as detected by Rrm3 accumulation

**Figure 5.**
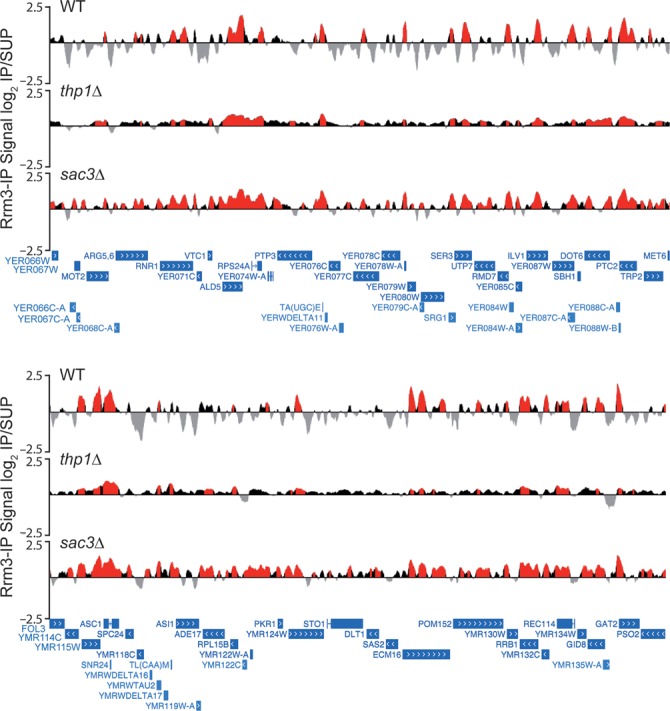
Genome-wide Rrm3 recruitment in *thp1Δ* and *sac3Δ* cells. Analysis of Rrm3-FLAG binding to the whole genome by ChIP-chip. Two genomic fragments from chromosomes V and XIII are shown, with enrichment values represented as the signal log_2_ IP/SUP ratios. Red histograms (online version) represent the significant Rrm3 binding clusters (*P* < 0.01, minimum run >100 bp, maximum gap <250 bp). Genes and other features are represented according to the SGD.

Genetic instability observed in several mRNA export mutants has been reported to be a consequence of global replication impairment. This has been shown by the genome-wide accumulation of the replicative Rrm3 helicase, which is required for the progression of RFs through obstacles in the DNA, including highly transcribed genes (43,44). To assess whether replication impairment occurs in TREX-2 mutants, ChIP-chip analyses were performed with an Rrm3-FLAG fusion protein in wild-type, *thp1Δ* and *sac3Δ* cells, elaborating a genomic map distribution. We found that Rrm3 was distributed all over the genome in the three analyzed strains (Supplementary Figure S8). However, as can be seen in Figure 5, Rrm3 clusters were more abundant in *sac3Δ* cells (7103 Rrm3 clusters versus 5261 in wild type; 5197 in *thp1Δ*). The low Rrm3-IP signal in *thp1Δ* cells may be due to a less efficient immunoprecipitation in this strain as a consequence of its growth defect, which would lead to a lower proportion of cells in S phase and thus a lower detection of Rrm3. Importantly, however, Rrm3 clusters were significantly longer in both mutants (average size of 736 bp in *sac3Δ* and 620 bp in *thp1Δ* versus 475 bp in wild type, *P* < 0.001), covering 32% of the genome in *sac3Δ* cells and 27% in *thp1Δ* versus 21% in the wild type. This indicates that replication obstacles accumulate in the absence of Sac3 and Thp1, this effect being stronger in *sac3Δ* cells, which lack the major subunit of the complex. Western analyses showed that this genome-wide increase in Rrm3 recruitment was not due to an increase in total Rrm3-FLAG protein levels (Supplementary Figure S9). In addition, the percentage of clusters mapping at ORFs was also higher in the mutants than in the wild type (87.6% in *sac3Δ* and 83% in *thp1Δ* versus 76.0% in the wild type), consistent with RF stalling occurring preferentially at transcribed genes and correlating with the distribution of Sac3 and Thp1 in these regions (Figures 2–4). The data are consistent with the accumulation of replication stalls in TREX-2 mutants, but the fact that double mutants *thp1Δ rrm3Δ* and *sac3Δ rrm3Δ* are viable (Supplementary Figure S10) suggests that Rrm3 is not essential for replication resumption.

**Figure 6.**
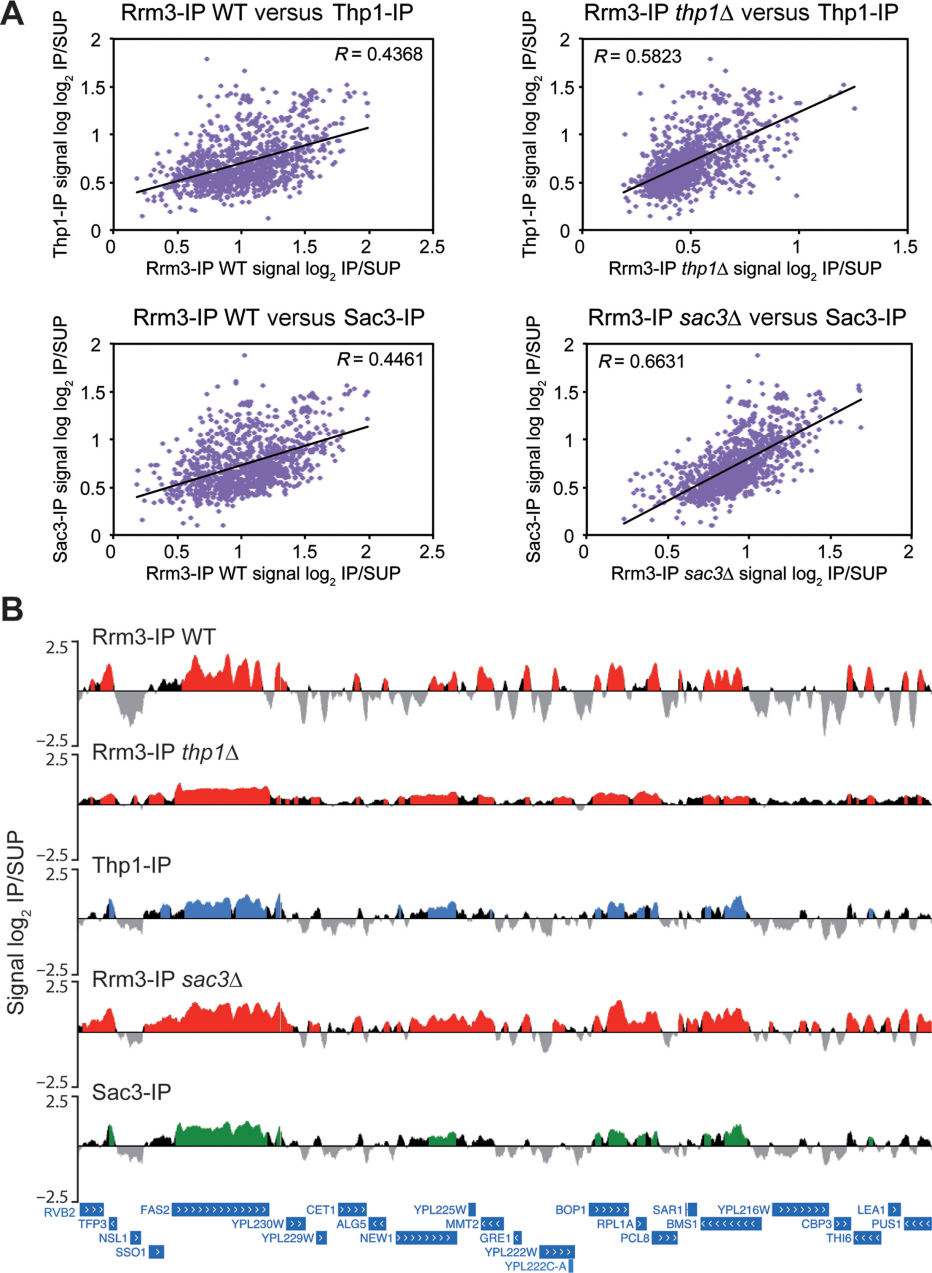
Analysis of Rrm3-enriched genes in wild-type, *thp1Δ* and *sac3Δ* cells. (**A**) Correlation analysis of Thp1, Sac3 and Rrm3 enrichment levels in *thp1Δ* and *sac3Δ* cells. Scatter plots showing correlation among Thp1, Sac3 and Rrm3 enrichment levels in the common ORFs detected by ChIP-chip in wild-type and mutant strains, represented as signal log_2_ IP/SUP ratio. A linear regression line and the Pearson's *R* correlation coefficient are plotted per graph. The complete list of genes can be found in the Supplementary Dataset II. (**B**) Similar distribution of Thp1, Sac3 and Rrm3 along the genome. Thp1, Sac3 and Rrm3 binding to a fragment of chromosome XVI detected by ChIP-chip and represented as signal log_2_ IP/SUP ratio is shown. Red (Rrm3-IP), blue (Thp1-IP) and green (Sac3-IP) histograms (online version) represent the statistically significant protein binding clusters (*P* < 0.01, minimum run >100 bp, maximum gap <250 bp). Genes and other features are represented according to the SGD.

Analysis of the ORFs mapped by Rrm3 revealed that it was enriched in the same genes in wild-type and mutant cells, plus a high number of additional ones in the mutants, specially in *sac3Δ* (3926 in the wild type versus 4190 genes in *thp1Δ* and 5520 genes in *sac3Δ*; Supplementary Figure S11A), indicating that replication impairment extends to more genes in the absence of Thp1 or Sac3. The enrichment levels of Rrm3 at the common genes were highly correlated between wild type and mutants (*R* = 0.5143 for *thp1Δ* and *R* = 0.7721 for *sac3Δ*; Supplementary Figure S11B). Interestingly, most genes associated with Thp1 or Sac3 overlapped those in which Rrm3 accumulates, especially with those of the mutants (Supplementary Figure S11C). Correlations of Thp1 and Sac3 enrichment in wild-type cells with Rrm3 enrichment in *thp1Δ* or *sac3Δ* cells, respectively, were higher than with Rrm3 enrichment in the wild type (Figure 6A), suggesting that in the absence of both TREX-2 subunits, Rrm3 accumulates at higher levels in the genes to which they bind. Consistently, Thp1 and Sac3 genomic binding profiles were similar to that of Rrm3, in particular in the mutants (Figure 6B). Taken together, these observations indicate that RF stalls preferentially at genes where Thp1 and Sac3 normally act.

### Rrm3 accumulates at highly transcribed genes in the absence of Sac3

**Figure 7.**
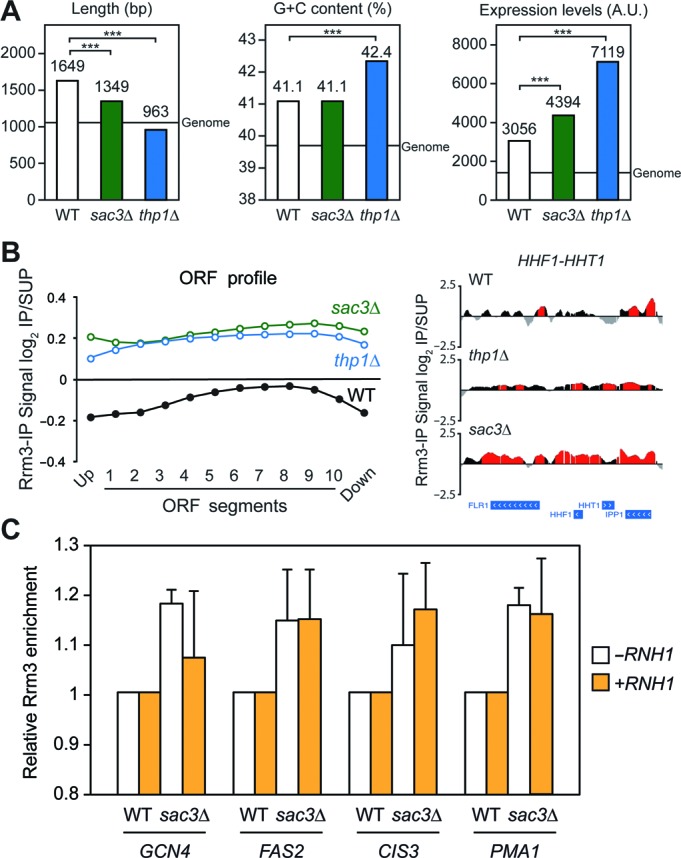
Rrm3 accumulation at highly transcribed genes in the absence of Thp1 and Sac3. (**A**) Functional and structural features of Rrm3-enriched genes. Statistical analysis of length, G+C content and model-based expression levels of the Top-500 genes showing significant Rrm3 enrichment in wild-type, *thp1Δ* and *sac3Δ* cells in ChIP-chip experiments. Median values are shown and a line represents the genome median. ***, *P* < 0.001 (Mann–Whitney's *U*-test) as compared to the wild type. The complete list of genes can be found in the Supplementary Dataset II. (**B**) Rrm3 accumulation at ORFs in *thp1Δ* and *sac3Δ* cells. Left, composite profile of Rrm3 occupancy detected by ChIP-chip across the average ORF, plotted as Rrm3 signal log_2_ IP/SUP ratio average per segment considering total genes, in wild-type, *thp1Δ* and *sac3Δ* cells. Right, Rrm3 distribution at the highly transcribed *HHF1-HHT1* locus in wild-type and *sac3Δ* cells, with enrichment values represented as the signal log_2_ IP/SUP ratio. Red histograms (online version) represent the statistically significant Rrm3 binding clusters (*P* < 0.01, minimum run >100 bp, maximum gap <250 bp). Genes and other features are represented according to the SGD. (**C**) Analysis of R-loop-dependence of Rrm3 accumulation in *sac3Δ* cells. Specific Rrm3-FLAG ChIP analysis using qPCR of four genes showing high Rrm3 enrichment levels in the ChIP-chip experiments, in WRBb-9B (WT) and S3RBb-5B (*sac3Δ*) strains carrying pCM184 (-*RNH1*) or pCM184RNH1 (+*RNH1*) without doxycycline. Data were normalized to wild-type levels. Average and standard error of the mean of three independent experiments are shown.

To determine the structural and functional differences between wild-type, *thp1Δ* and *sac3Δ* cells in the genes in which Rrm3 accumulates, we selected the top 500 ORFs with the highest signal log_2_ ratio average in a sliding window of 200 bp, as detected in the ChIP-chip experiments. Statistical analyses revealed that these genes were significantly longer (except for *thp1Δ*), G+C-richer and more expressed than the genome median (*P* < 0.001; Figure 7A), consistent with previous results (29,30). Interestingly, genes in which Rrm3 accumulates at the highest level in *thp1Δ* and *sac3Δ* cells were more expressed than those of the wild type (*P* < 0.001; Figure 7A), suggesting that replication stalling preferentially occurs at the highest transcribed genes in the absence of Thp1 and Sac3. This conclusion is also supported by the correlation between Rrm3 enrichment and expression levels in the 3918 genes enriched in Rrm3 that are common between wild-type and *sac3Δ* cells (*R* = 0.4106 *vs.* 0.2289 in the wild type; Supplementary Figure S12), and in the 3394 genes that are common between wild-type and *thp1Δ* cells (*R* = 0.6313; Supplementary Figure S12). In addition, gene ontology analyses revealed an overrepresentation of genes involved in processes such as gene expression, transport and ribosome biogenesis (Supplementary Dataset I), consistent with those genes being highly transcribed in yeast.

Next, the relative distribution of Rrm3 along the length of the genes was analyzed, as explained below, using the ChIP-chip data. We found that Rrm3 distribution in wild-type cells increased towards the 3′ end of the genes, being non-detectable or low abundant in the promoter and terminator regions. This accumulation was similar in *thp1Δ* cells and lower in *sac3Δ* cells, suggesting that RF stalling occurs more homogeneously throughout the length of the genes in *sac3Δ* cells (Supplementary Figure S13A). This observation remained the same when genes of different length intervals were plotted (Supplementary Figure S13B). Importantly, we found that the average Rrm3 enrichment levels along the ORFs were higher both in *thp1Δ* and *sac3Δ* cells (Figure 7B), indicating that replication obstacles in genes are more abundant in the mutants. We also found Rrm3 enrichment in *thp1Δ* and *sac3Δ* cells at centromeres, in some of which Sac3 could also be seen in wild-type cells (Supplementary Figure S4), as well as enrichment in ARSs, introns, snRNAs, snoRNAs and RNAPIII-transcribed genes, including tRNAs (Table 1), with the average Rrm3 levels around centromeres, tRNAs and snoRNAs being higher in the mutants (Supplementary Figure S14). Altogether, these results indicate that replication obstacles accumulate preferentially at the highest transcribed genes when Thp1 or Sac3 are absent and extend to more regions than in the wild type.

Finally, since R loops have been suggested as potentially responsible for the genetic instability phenotypes of TREX-2 mutants (26), we analyzed whether replication stalling was linked to R-loop accumulation in *sac3Δ* cells. To assess this, we performed specific Rrm3-FLAG ChIP qPCR experiments in selected genes showing high levels of Rrm3 accumulation in the ChIP-chips. As can be seen in Figure 7C, the high levels of Rrm3 observed in *sac3Δ* cells in these genes were unaffected by RNase H1 overexpression, suggesting that replication stalls detected by Rrm3 are not strictly linked to R-loop accumulation in the sites analyzed.

## DISCUSSION

The factors involved in coupling transcription and mRNA export have been largely studied, although little is known about their roles at the genomic level. Here we show by a genome-wide approach that the Thp1 and Sac3 subunits of the yeast TREX-2 complex are involved in the transcriptional control of a large amount of genes, which is better observed in those highly transcribed. They bind to these genes and prevent transcription–replication collisions all over the genome, as determined by the accumulation of Rrm3 in the absence of these proteins. These findings make TREX-2 a relevant player in transcription that helps prevent transcription-associated genome instability all over the genome.

### Genome-wide function of Thp1–Sac3 at coding and non-coding transcribed genes

The yeast TREX-2 complex is an important mediator of mRNA export whose mutations lead to mRNA export defects and transcription elongation impairment (12,13,15,16,26,27). Here we show that *THP1* and *SAC3* deletions have a similar effect over gene expression, consistent with Thp1 and Sac3 working as a functional and structural unit (12,18) (Figure 1). Thus, down-regulated genes in TREX-2 mutants are long, G+C-rich and highly expressed, independently of their function. This is consistent with the transcription impairment observed in TREX-2 mutants using artificial reporters (12,26,27,32), and similar to the features of down-regulated genes in THO mutants (29), suggesting a common mechanism by which suboptimal mRNPs may have a feedback effect on transcription. However, functional differences may exist between THO and TREX-2, as suggested by the clustering and correlation analyses and by previous results. First, the THO complex is believed to act at the first step of the mRNP biogenesis and export pathway, while TREX-2 functions later, close to the NPC (3). Second, overexpression of the export factors Sub2 and Nab2 has different effects on THO and TREX-2 mutants (12,26,34). Thus, it is likely that the differences observed in the global gene expression analyses may reflect a different impact of THO and TREX-2 mutations over transcription and mRNA export. On the other hand, depletion of Sus1, a TREX-2 component that is also part of the H2B deubiquitinylation module of the SAGA complex, leads to a transcriptional profile clearly different from those of THO, *thp1Δ* and *sac3Δ* mutants. This is consistent with a differential effect of TREX-2 mutations in transcription elongation (27).

Previous studies have shown that Thp1, as well as the module formed by Sac3, Thp1 and Sem1, binds nucleic acids *in vitro* (12,18). Despite this, recruitment of Thp1 and/or Sac3 to the chromatin was not previously reported. Here we show that Thp1 and Sac3 bind to transcribed genes *in vivo*, especially to those long, G+C-rich and highly transcribed (Figures 2–4), according to the features of down-regulated genes in the mutants. This supports a global function of Thp1 and Sac3 in highly transcribed genes. Consistently, several mRNA export factors and NPC components in yeast also associate with transcriptionally active genes (29–31,45,46). Moreover, a recent report showed that human cells depleted of GANP, the Sac3 homolog, had a defect in the export of highly expressed transcripts (47). The genes enriched in Thp1 and Sac3 are also bound by the THO subunit Hpr1, consistent with the idea that both complexes act in the same process. The fact that Hpr1 is enriched in more genes than Thp1 and Sac3 could be due to different detection efficiencies of these proteins, caused by either a lower efficiency of antibody recognition of TREX-2 subunits or a lower stability of Thp1 and Sac3 proteins.

We found that Thp1 and Sac3 accumulate towards the 3′ end of the genes (Figure 4), consistent with their function in transcription elongation and RNA metabolism (26,27). This distribution does not depend on the length of the nascent mRNA and is similar to the 3′-end accumulation of phosphorylated Ser2 in the C-terminus domain (CTD) of the RNAPII, a mark of transcription elongation (48,49). Similar binding distributions have been previously reported for transcription elongation and mRNA export factors (29–31,49). Recruitment of Thp1 and Sac3 to genes might depend on elongation factors or might occur through direct RNA binding or via an indirect interaction with other proteins. One candidate could be the export receptor Mex67-Mtr2, given the interaction of Sac3 with this factor (13). In addition, TREX-2 might play a role in the coordination of 3′-end processing steps, similarly to THO and Sub2 (50,51), contributing to mRNA polyadenylation and/or release from the transcription termination site.

Apart from coding genes, TREX-2 proteins bind to other transcribed sequences, such as snRNAs, snoRNAs and RNAPIII-transcribed genes, including tRNAs (Table 1). This observation is consistent with that observed for other mRNA export factors, such as THO, Npl3 and Nab2 (29,30,52,53), and abrogates for a general function of these proteins in transcription not restricted to coding regions or RNAPII genes. This would imply that TREX-2 functions in the export of RNAPII and non-RNAPII-generated transcripts. Additionally, Sac3 was also found at centromeres (Table 1). This might imply an additional function of TREX-2 related to chromosome segregation and cell division. Indeed, Sac3 and Cdc31, as well as another mammalian Sac3-homolog called Shd1, are required for the duplication of either the spindle pole bodies or centrosomes (25,54,55).

### Global function of TREX-2 preventing transcription–replication conflicts

Transcription is known to constitute an obstacle for RF progression and improper transcription and mRNP biogenesis can lead to replication impairment and genome instability (56). Here we show that the replicative Rrm3 helicase, which is required for the RF to pass through obstacles in the DNA, such as transcribed areas (43,44), is strongly accumulated at highly expressed genes in *thp1Δ* and *sac3Δ* cells (Figures 5–7). As Thp1 and Sac3 also accumulate in these genes and *thp1Δ* and *sac3Δ* mutants are affected in transcription, we propose that the RF progresses less efficiently through highly transcribed regions due to the lack of either Thp1 or Sac3, leading to the detected replication impairment. These data agree with the TAR phenotype of TREX-2 mutants (12,26,32). It is worth noting that changes in the Rrm3 presence in *sac3Δ* cells is not linked to R-loop accumulation at the four sites analyzed. This result resembles the situation of human cells depleted of TREX-2 components, which does not accumulate detectable levels of R loops (57). However, as it is known that RNase H1 overexpression does not completely suppress the genome instability phenotypes of THO and TREX-2 mutants (26,58), it seems clear that not all events related to genome instability are mediated by R-loops in TREX-2 mutants, even though they are mediated by the RNA molecule (26). An interesting possibility to explore in the future would be whether this phenomenon is mainly related to a different anchoring of transcribed genes to NPCs in these mutants (59). This could be consistent with an early role of THO in RNA metabolism and a later role of TREX-2, as deduced from the different protein composition of the mRNPs depending on whether they are purified using 5′-end RNA processing proteins versus NPC components (60).

In addition to coding genes, replication obstacles extend to other regions, in agreement with Thp1–Sac3 distribution. Similar Rrm3 accumulation has been detected in other mRNA-processing mutants, such as *hpr1Δ* or *npl3Δ* (29,30). Furthermore, anchoring of genes to the NPC during transcription may generate topological constraints that could constitute an obstacle for replication. In agreement with this idea, it has been proposed that the DNA damage checkpoint in yeast is required for the release of transcribed genes from the NPC to facilitate RF progression, since mutations in THO, TREX-2 and some nucleoporins rescue the RF defects of DNA-damage checkpoint mutants (59). In contrast, our results indicate that the absence of Thp1 or Sac3 in cells with an intact checkpoint leads to an increase of replication stalls.

Therefore, this work provides evidence of a function of TREX-2 complex at the genome-wide level, especially at highly expressed genes, and supports the current model that improper mRNP biogenesis can lead to global replication impairment and genome instability. Further work will be required to decipher the molecular nature of these events and the role of the nascent mRNA. Given the high conservation of TREX-2, It would also be interesting to study whether similar mechanisms act in higher eukaryotes.

## ACCESSION NUMBERS

Microarray and ChIP-chip data can be accessed at Gene Expression Omnibus (GSE56703, GSE56702, GSE56700).

## SUPPLEMENTARY DATA

Supplementary Data are available at NAR Online.

SUPPLEMENTARY DATA
