# Supplementary material for: A genome-wide function of THSC/TREX-2 at active genes prevents transcription–replication collisions
Source: Nucleic Acids Res. 2014 Oct 7;42(19):12000–14. doi: 10.1093/nar/gku906 (PMC4231764; doi:10.1093/nar/gku906)
Supplement: SUPPLEMENTARY DATA [file supp_42_19_12000__index.html]

A genome-wide function of THSC/TREX-2 at active genes prevents transcription–replication collisions — SUPPLEMENTARY DATA 

# A genome-wide function of THSC/TREX-2 at active genes prevents transcription–replication collisions

## SUPPLEMENTARY DATA

**Files in this Data Supplement:**

- SUPPLEMENTARY DATA
