# Supplementary material for: A genome-wide function of THSC/TREX-2 at active genes prevents transcription–replication collisions
Source: Nucleic Acids Res. 2014 Oct 7;42(19):12000–14. doi: 10.1093/nar/gku906 (PMC4231764; doi:10.1093/nar/gku906)
Supplement: SUPPLEMENTARY DATA [file supp_gku906_nar-01412-x-2014-File009.zip › NAR-01412-X-2014.R1 Suppl files/Santos-Pereira_Supplementary.pdf]

SUPPLEMENTAL DATA

A genome-wide function of THSC/TREX-2 at active genes prevents transcription-replication collisions

José M. Santos-Pereira, María L. García-Rubio, Cristina González-Aguilera, Rosa Luna and Andrés Aguilera *

Centro Andaluz de Biología Molecular y Medicina Regenerativa CABIMER, Universidad de Sevilla-CSIC, Seville, Spain

* To whom correspondence should be addressed. . *E-mail*: aguilo@us.es

Inventory: **14 Supplemental Figures and Legends**

3 Supplemental Tables

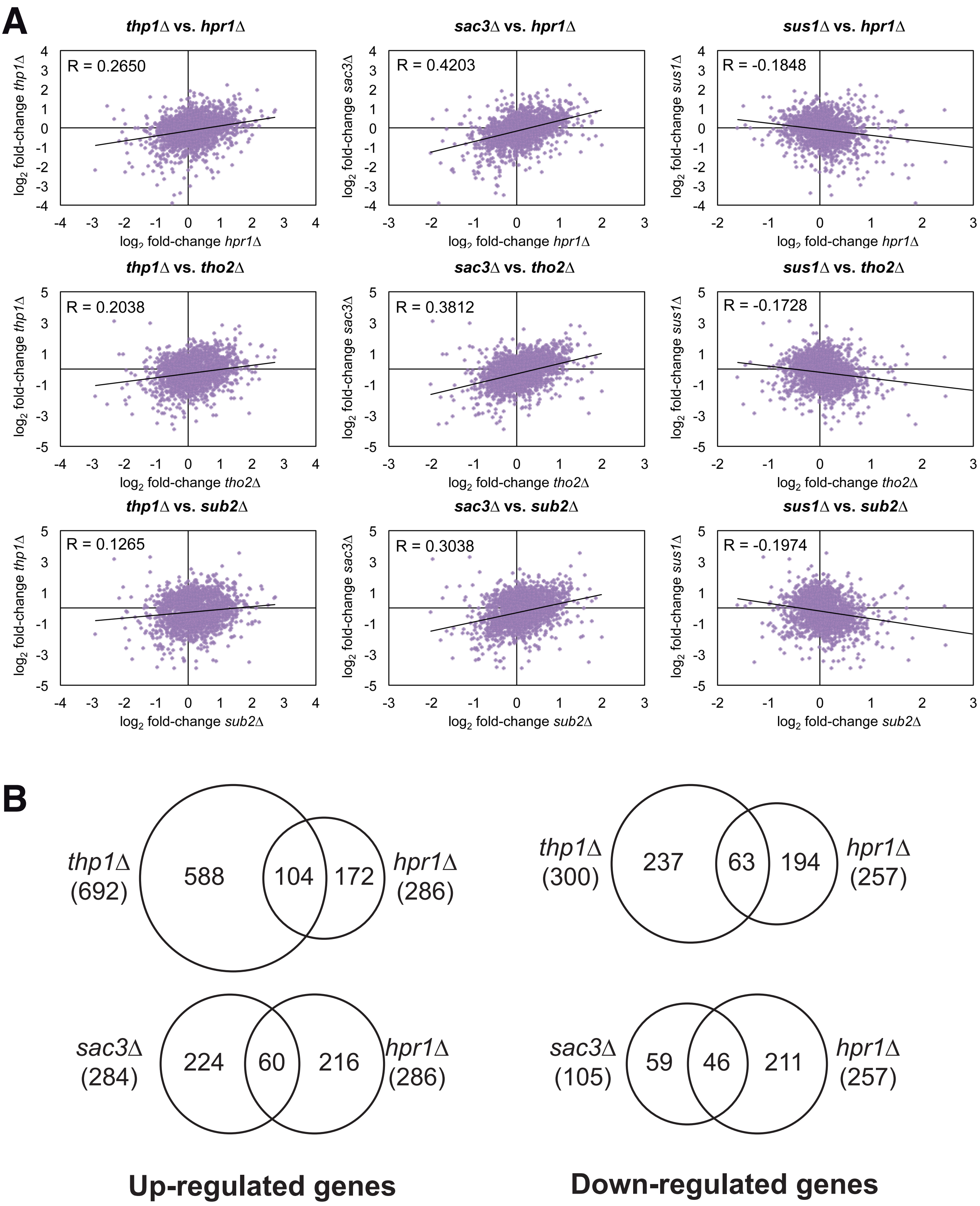

Figure S1 – Comparison of changes in gene expression in THO, Sub2 and THSC/TREX-2 mutants.
(A) Scatter plots showing correlation among the expression profiles of THO, Sub2 and THSC/TREX-2 mutants.
(B) Venn diagrams showing the overlap between up- and down-regulated genes in *thp1Δ*, *sac3Δ* and *hpr1Δ* mutants. See Figure 1 for other details.

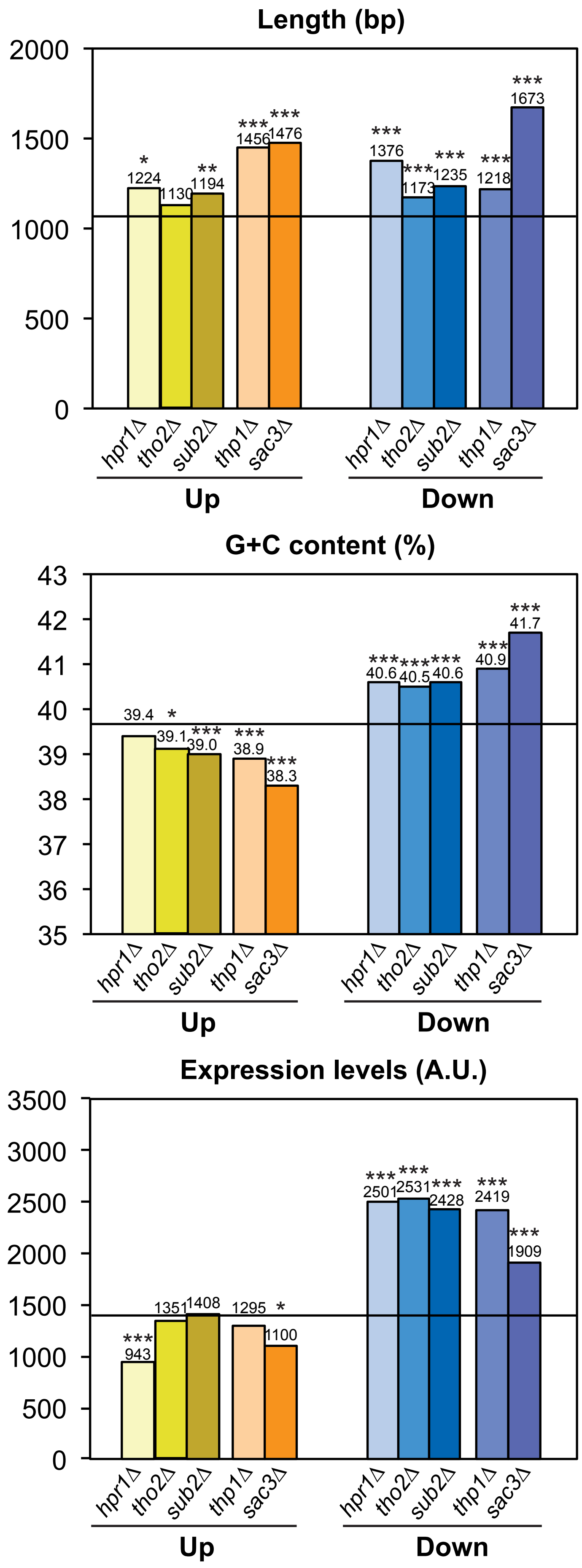

Figure S2 – Structural and functional features of the genes whose expression is affected in THO, Sub2 and THSC/TREX-2 mutants.
Statistical analysis of length, G+C content and model-based expression levels of genes whose expression levels are affected in THO, Sub2 and THSC/TREX-2 mutants. *, P<0.05, **, P<0.01, ***, P<0.001 (Mann-Whitney's U-test) compared to genome median. See Figure 1 for other details.

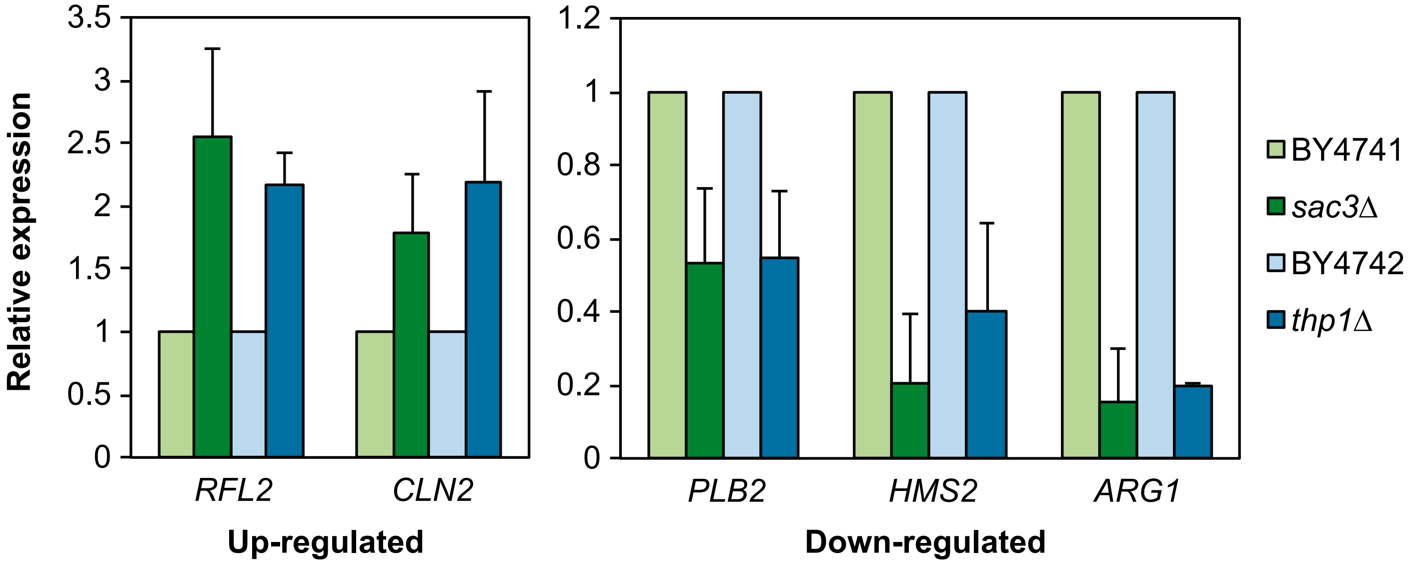

Figure S3 – Validation of changes in gene expression in *thp1Δ* and *sac3Δ* cells
RT-qPCR analysis of gene expression in BY4741 (WT), Y03517 (*sac3Δ*), BY4742 (WT) and Y11764 (*thp1Δ*) cells, for two up-regulated (left) and three down-regulated genes (right). Average and standard error of the mean are shown for three independent experiments normalized to wild-type levels.

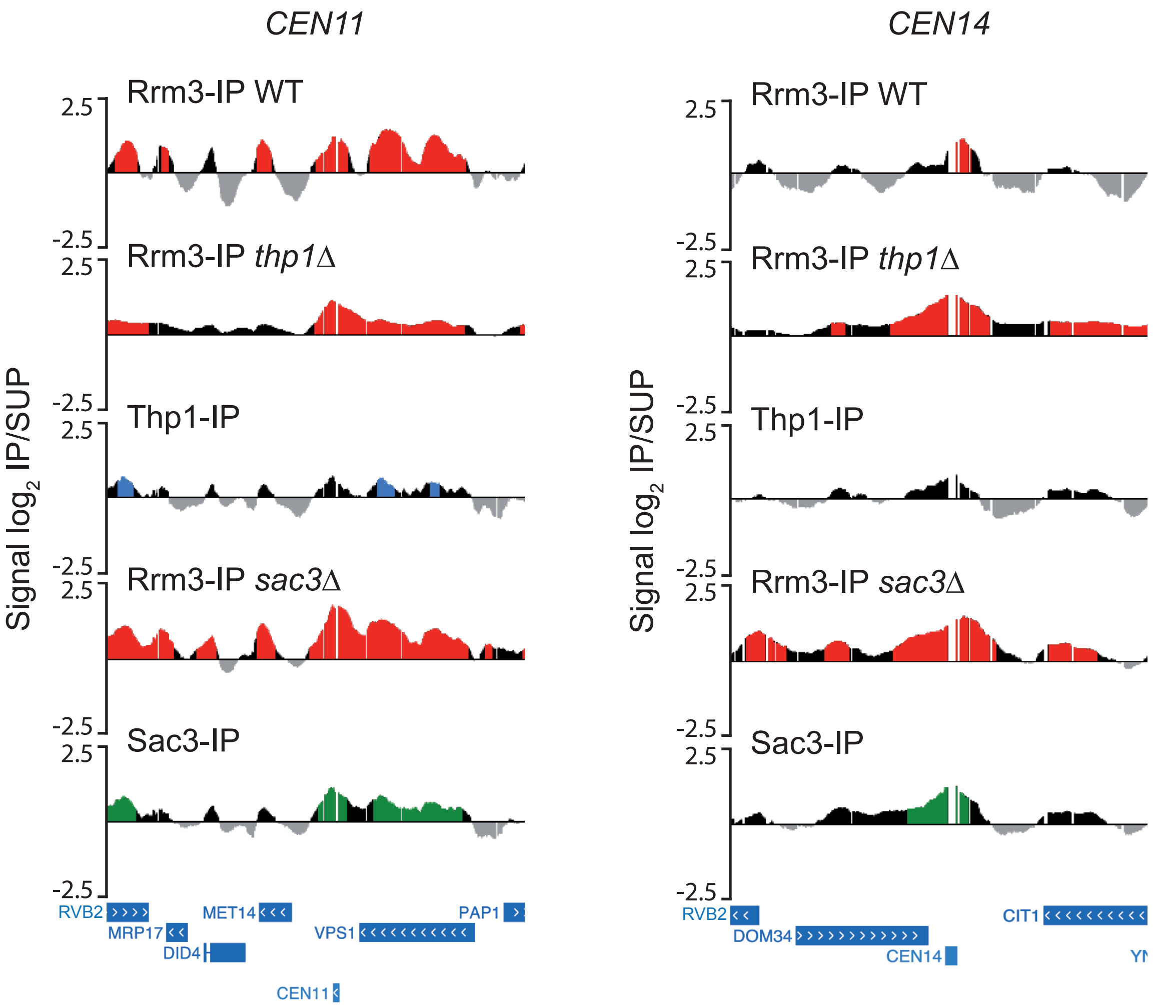

Figure S4 - Thp1, Sac3 and Rrm3 distribution around the centromeres. *CEN11* and *CEN14* regions are plotted. See Figure 6 for other details.

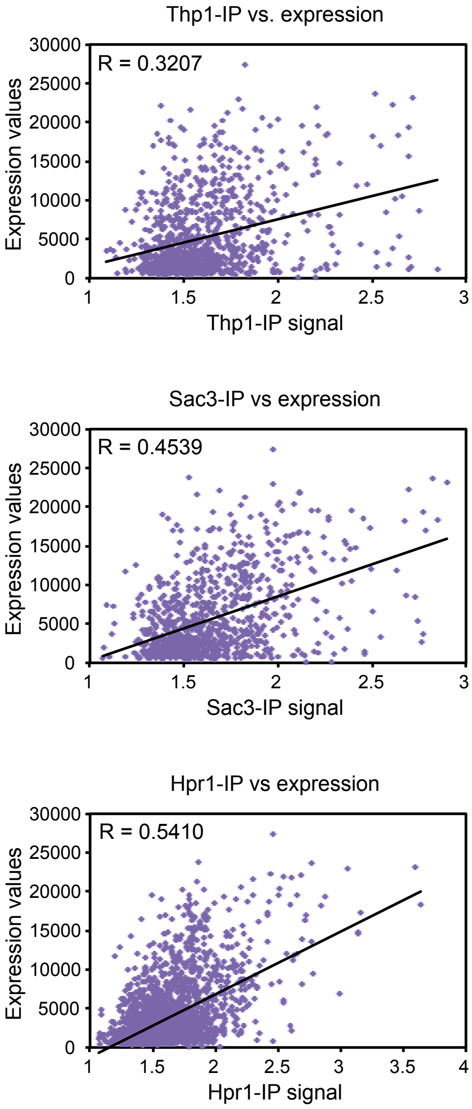

Figure S5 – Correlation between Thp1-, Sac3- and Hpr1-enrichment levels.
Scatter plots showing correlation among Thp1-, Sac3- and Hpr1-enrichment levels to the common mapped genes and their model-based expression values. See Figure 1 for other details.

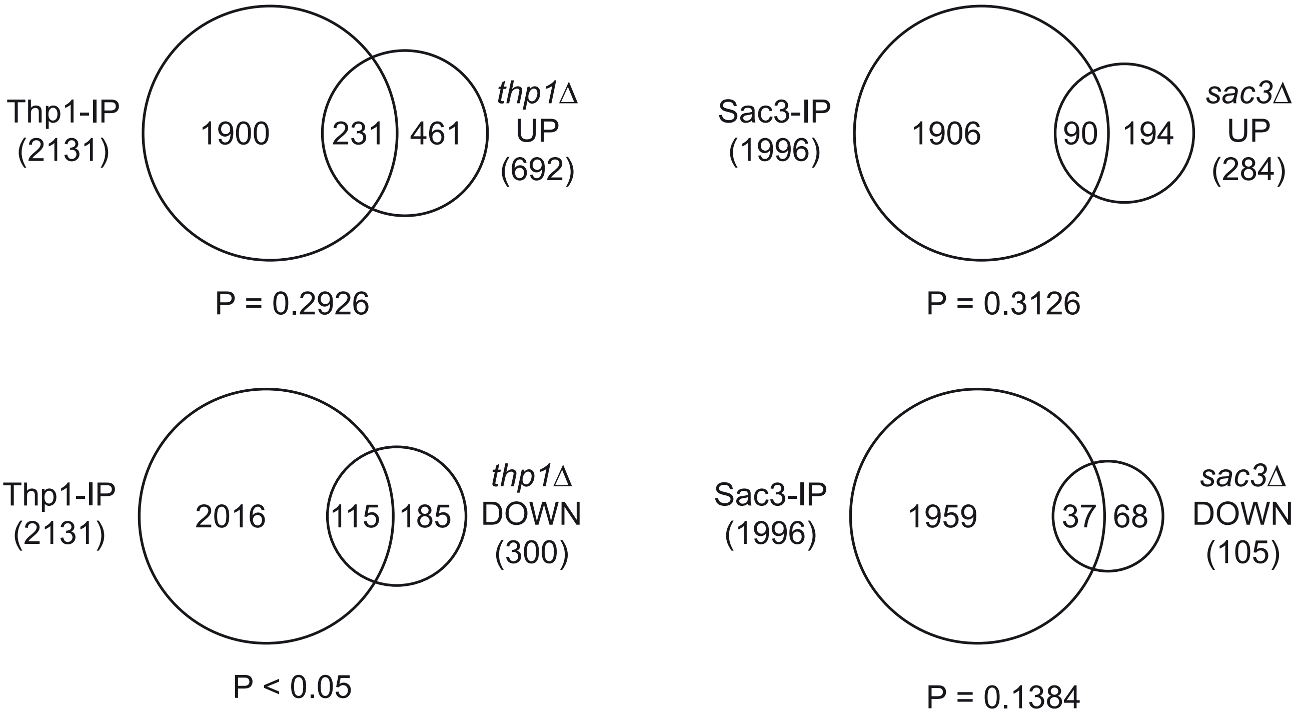

Figure S6 – Overlap between Thp1- and Sac3-bound genes and de-regulated genes in the mutants

Venn diagrams showing the overlap between Thp1- (left) and Sac3-bound genes (right) with up- (top) and down-regulated genes (bottom) in *thp1Δ* and *sac3Δ* cells

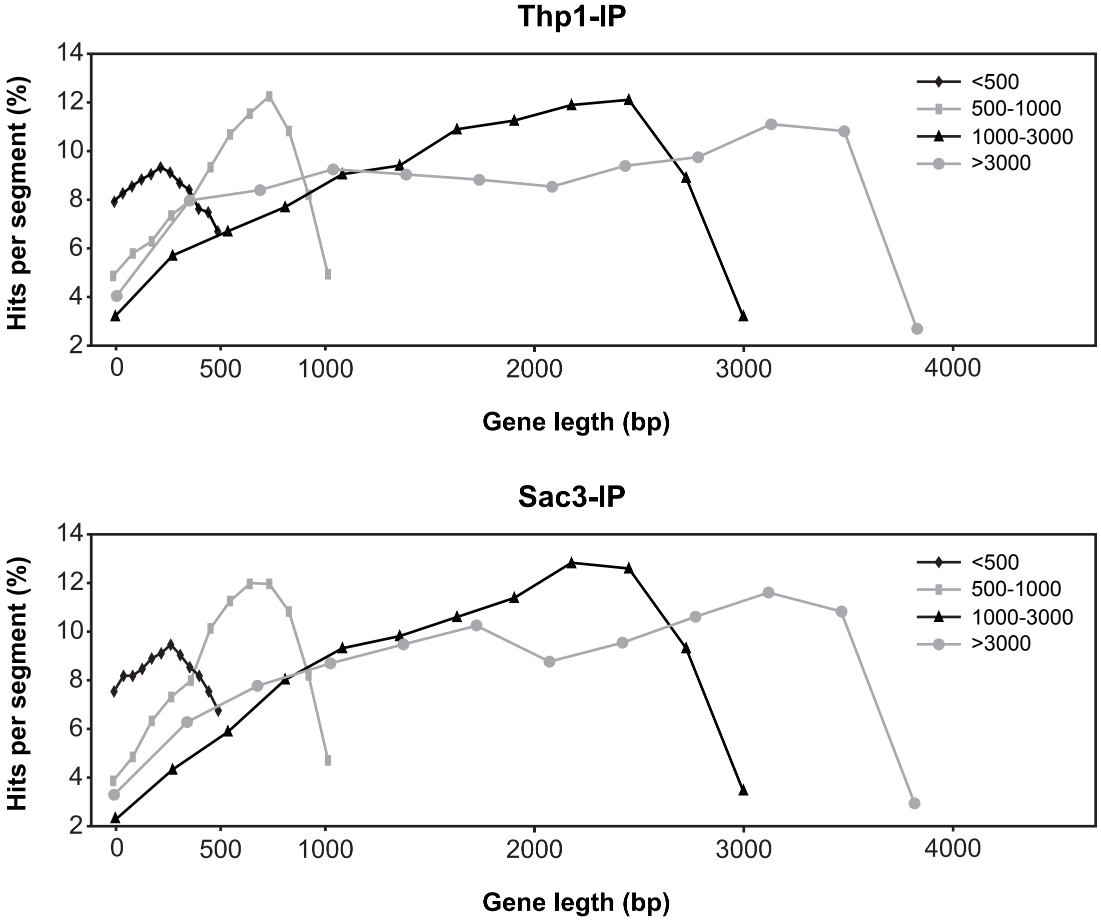

Figure S7 – Thp1 and Sac3 accumulate towards the 3' end of genes independent of length. Composite profiles of Thp1 and Sac3 occupancy across the average ORF for different intervals of gene length (see Materials and Methods).

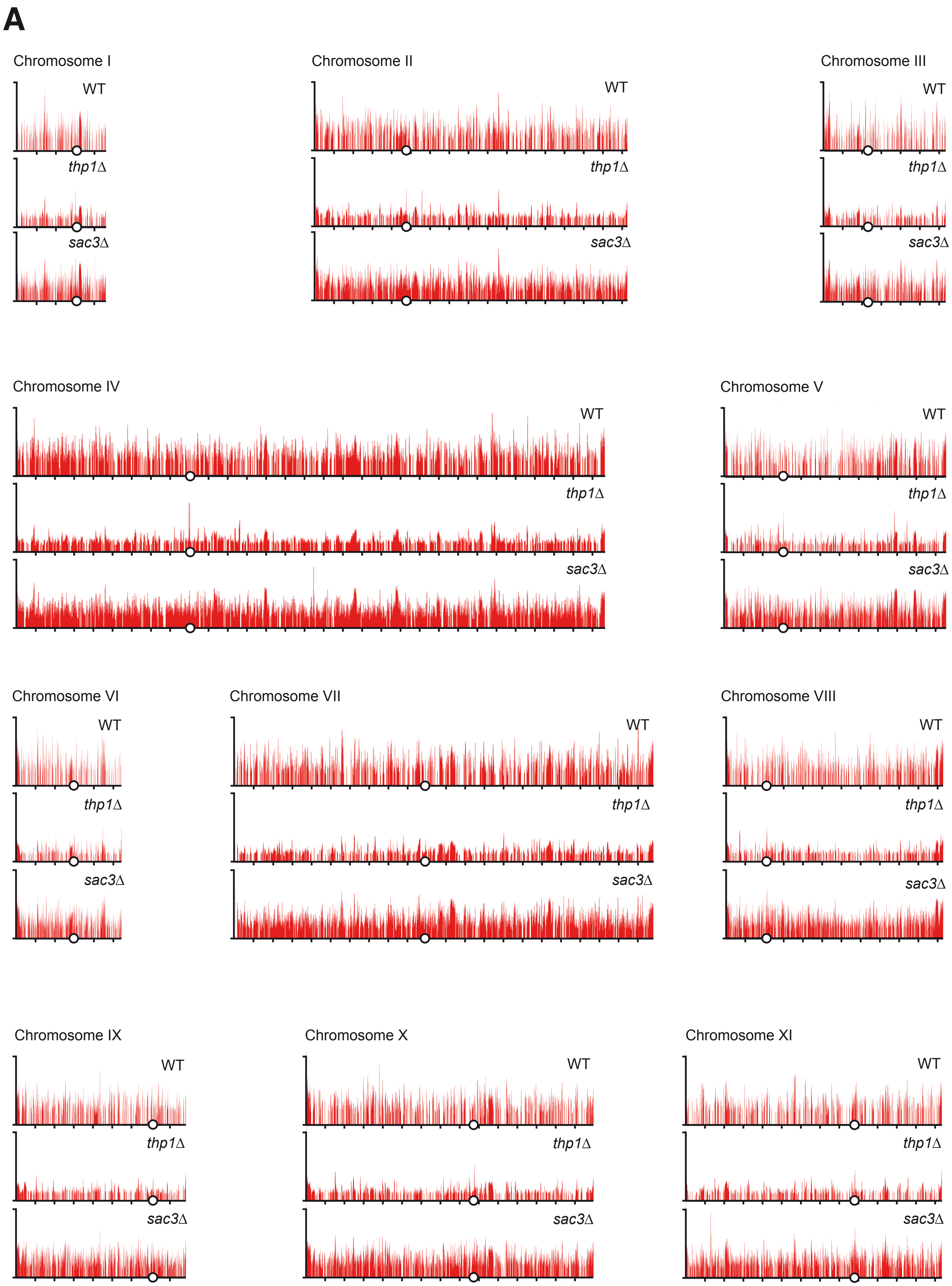

Figure S8 – Genomic view of Rrm3 enrichment in wild type, *thp1Δ* and *sac3Δ* cells.
Representations of chromosomes with the signal log_2 ratio values for the significant ChIP-chip clusters are plotted. The X-axis shows chromosomal coordinates in kb. Positions of centromeres are indicated as open circles.

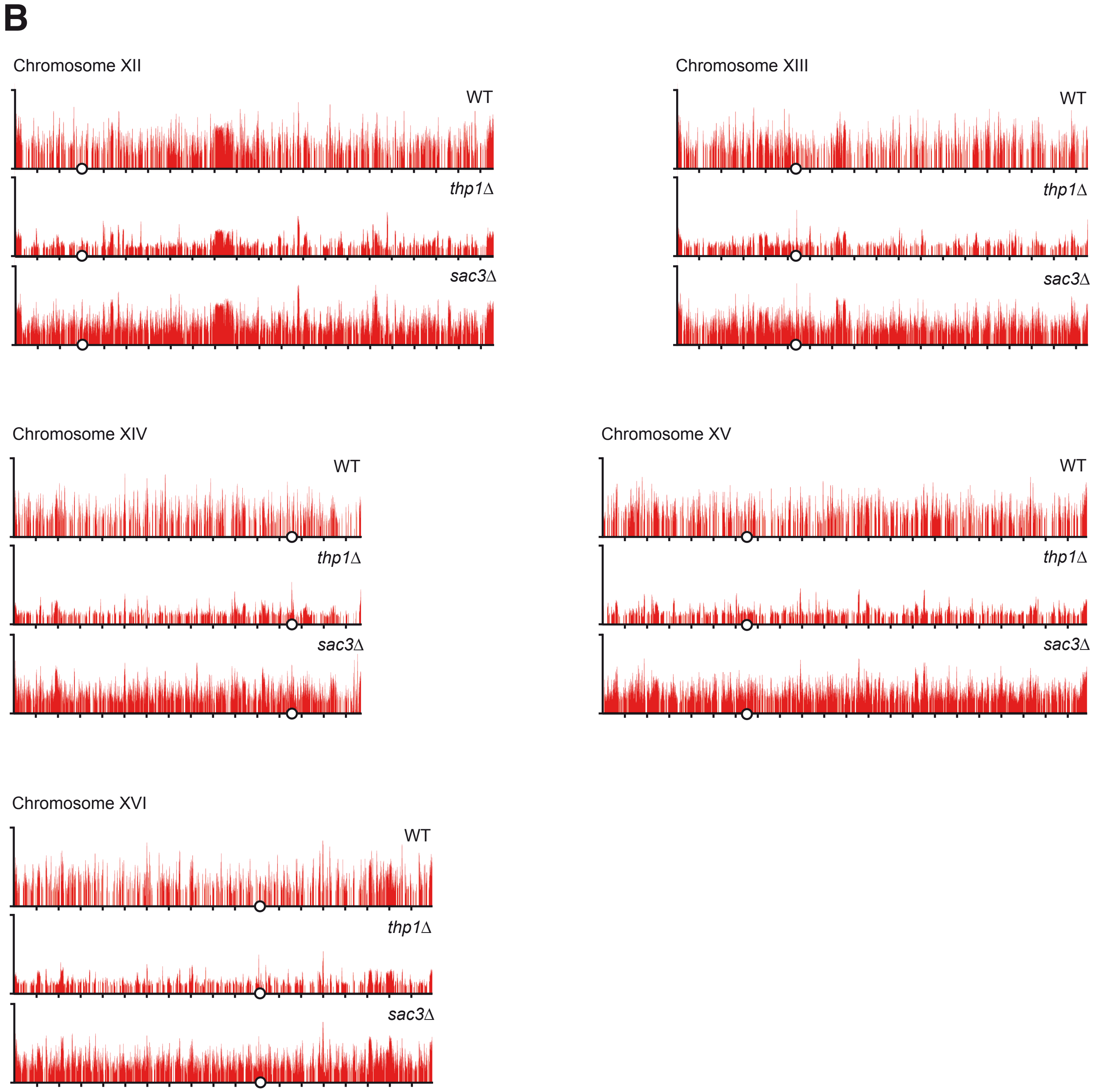

Figure S8 – Genomic view of Rrm3 enrichment in wild type, *thp1Δ* and *sac3Δ* cells.
Representations of chromosomes with the signal log_2 ratio values for the significant ChIP-chip clusters are plotted. The X-axis shows chromosomal coordinates in kb. Positions of centromeres are indicated as open circles.

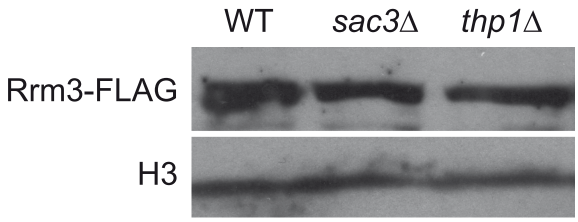

Figure S9 - Rrm3 protein levels are similar in the analyzed strains.
Western blot showing the levels of Rrm3-FLAG protein and histone H3 used as loading control in WRBb-9B (WT), S3RBb-5B (*sac3Δ*) and T1RBb-39A (*thp1Δ*) cells.

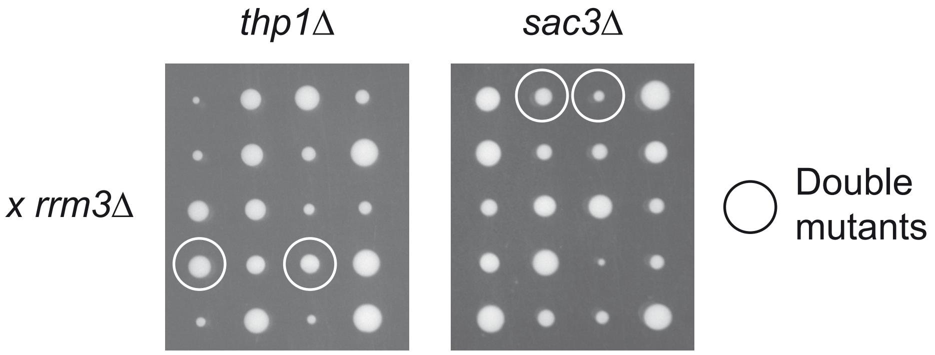

Figure S10 - *thp1Δ rrm3Δ* and *sac3Δ rrm3Δ* double mutants are viable
Tetrad analysis of genetic crosses among WRT1B1-4D (*thp1Δ*), WRS3B1-1C (*sac3Δ*) and WRRM3α-2B (*rrm3Δ*) cells.

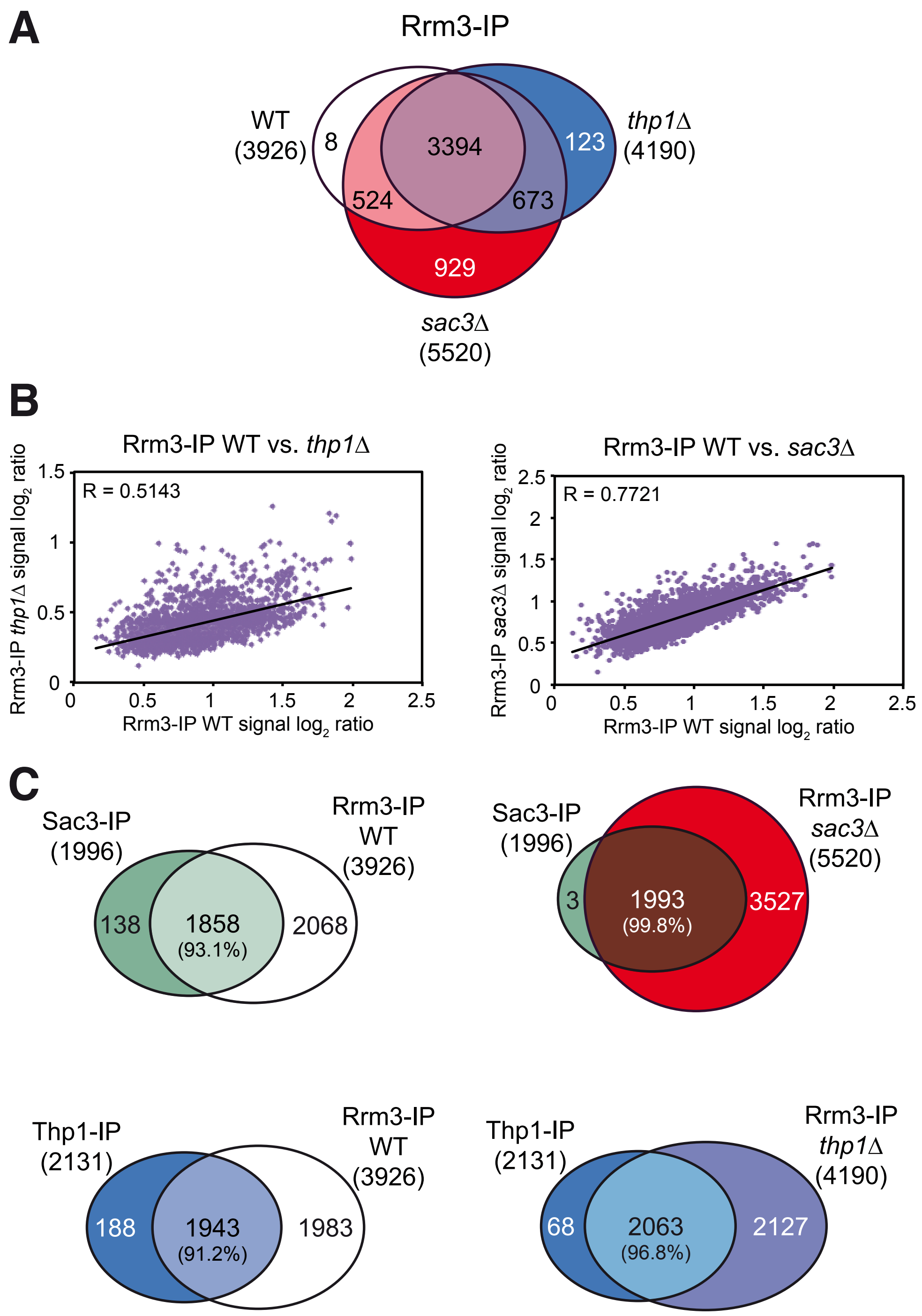

Figure S11 – Analysis of Rrm3-enriched genes in wild-type, *thp1Δ* and *sac3Δ* cells
(A) Venn diagram showing the overlap among Rrm3-enriched genes in wild-type, *thp1Δ* and *sac3Δ* cells. (B) Scatter plots showing correlation between Rrm3 enrichment levels in the common ORFs between wild-type and *thp1Δ* or *sac3Δ* cells. (C) Venn diagrams showing the overlap among Thp1-, Sac3- and Rrm3-bound genes.

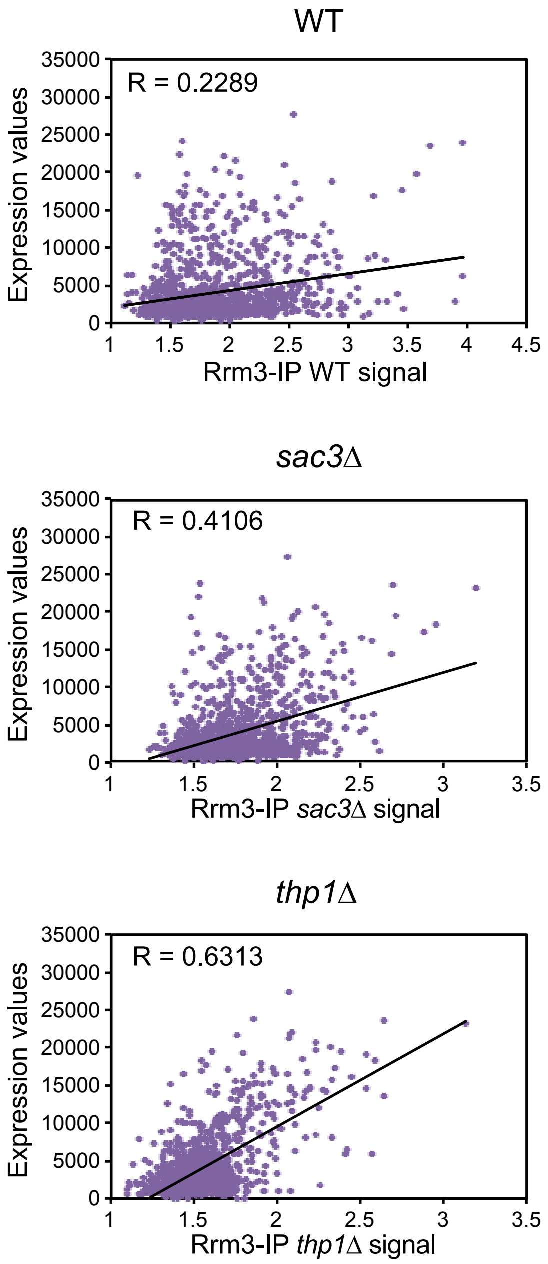

Figure S12 – Correlation between Rrm3 enrichment and expression levels.
Correlation between Rrm3 enrichment levels and model-based expression values in the genes with significant Rrm3 enrichment both in wild-type and *sac3Δ* or *thp1Δ* cells. See Figure 1 for other details.

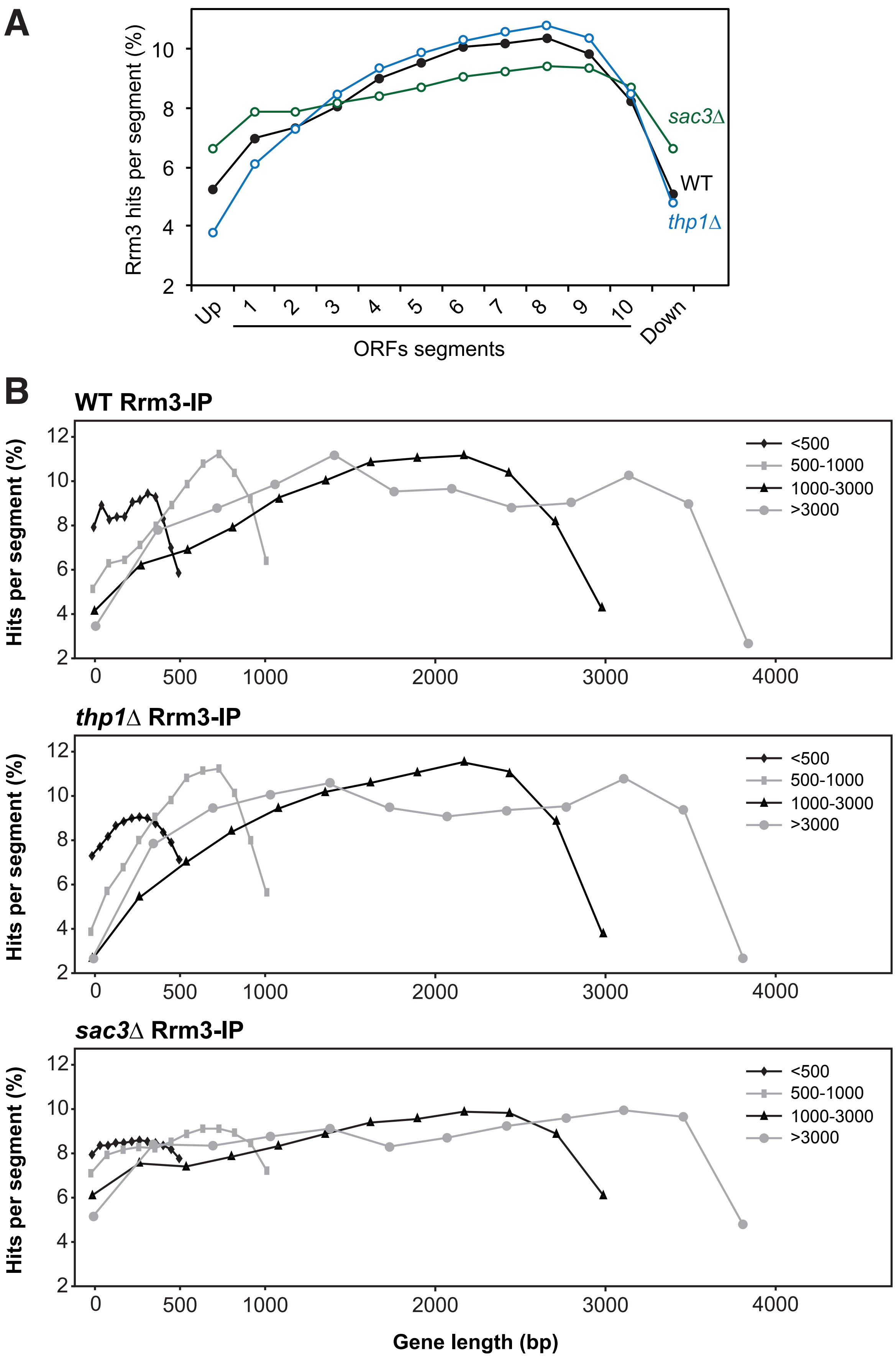

Figure S13 – Rrm3 distribution along the genes.
Composite profile of Rrm3 occupancy detected by ChIP-chip across the average ORF, plotted as Rrm3 percentage of ChIP clusters per segment (see Materials and Methods), in wild-type, *thp1Δ* and *sac3Δ* cells, considering **(A)** all ORFs or **(B)** ORFs with different gene lengths.

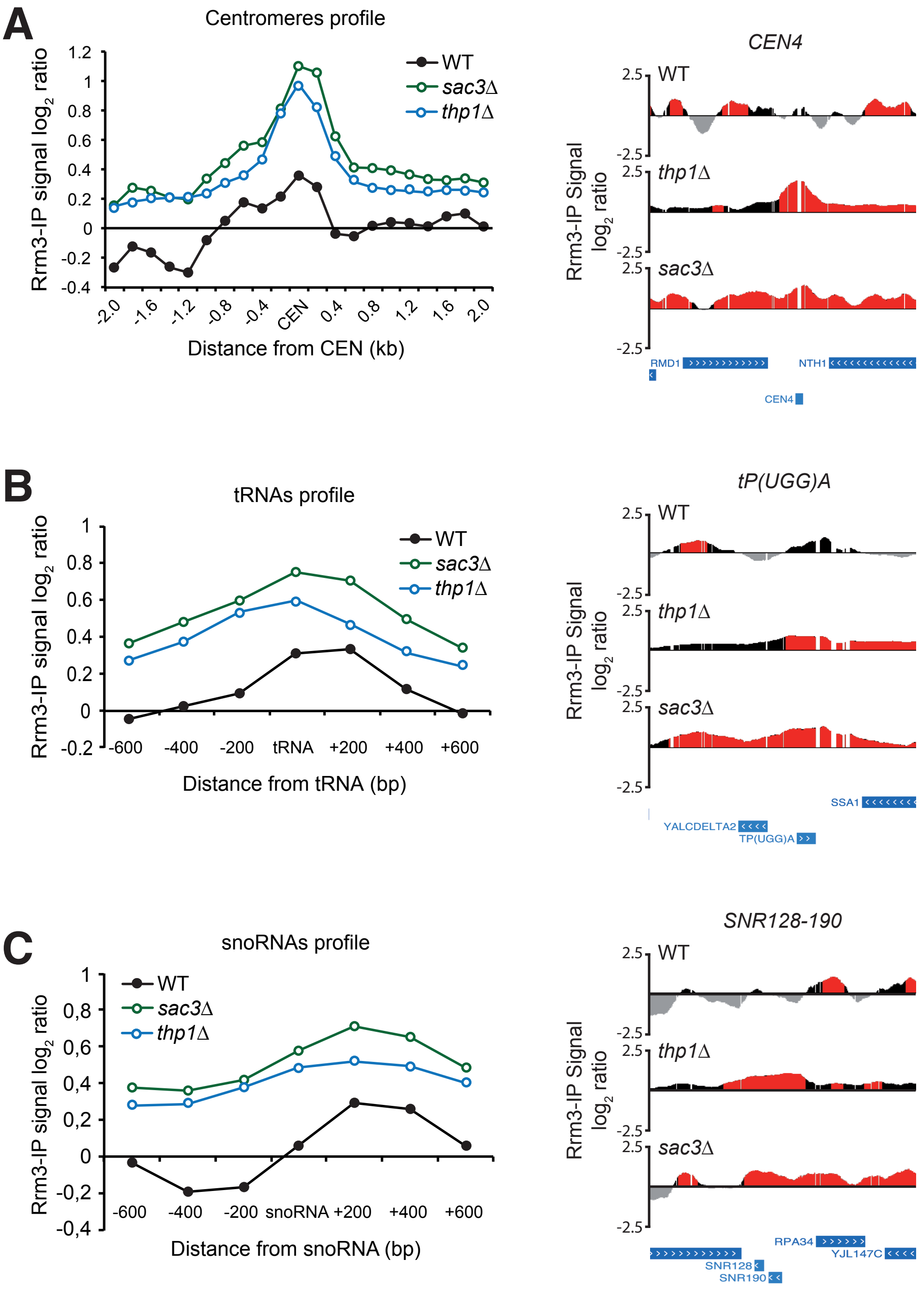

Figure S14 – Rrm3 is enriched in *sac3Δ* and *thp1Δ* cells in other genomic regions rather than genes.
Composite profile of Rrm3 occupancy detected by ChIP-chip across **(A)** 4-kb pericentromeric regions, **(B)** and 1.2-kb around tRNAs **(C)** and snoRNAs. Values are plotted as signal log_2 ratio average per segment considering total features in each case.

Table S1 – Genes up- or down-regulated in the analyzed THO-TREX and THSC/TREX-2 mutants.

Mutant	Up-regulated	Down-regulated	Total
hpr1Δ	286	257	543
tho2Δ	186	525	711
sub2Δ	291	621	912
thp1Δ	692	300	992
sac3Δ	284	105	389
sus1Δ	166	67	233

Table S2 - Yeast strains used in this study.

**Strains**	**Genotype**	**Source**
BY4741	*MAT*<u>**a**</u> *his3Δ1 leu2Δ0 met15Δ0 ura3Δ0*	Euroscarf
BY4742	*MAT*α *his3Δ1 leu2Δ0 lys2Δ0 ura3Δ0*	Euroscarf
Y03517	BY4741 *sac3Δ::KanMX*	Euroscarf
Y11764	BY4742 *thp1Δ::KanMX*	Euroscarf
Y17455	BY4742 *sus1Δ::KanMX*	Euroscarf
WT1F-04	*MAT*<u>**a**</u> *ade2-1 can1-100 his3-11,15 leu2-3,112 THP1::FLAG::KanMX trp1-1 ura3-1*	This study
WS3F-04	*MAT*<u>**a**</u> *ade2-1 can1-100 his3-11,15 leu2-3,112 SAC3::FLAG::KanMX trp1-1 ura3-1*	This study
WRT1B1-4D	*MAT*<u>**a**</u> *ade2-1 bar1Δ can1-100 his3-11,15 leu2-3,112 thp1Δ::KanMX trp1-1 ura3-1*	This study
WRS3B1-1C	*MAT*<u>**a**</u> *ade2-1 bar1Δ can1-100 his3-11,15 leu2-3,112 sac3Δ::KanMX trp1-1 ura3-1*	This study
WRRM3α-2B	*MAT*α *ade2-1 can1-100 his3-11,15 leu2-3,112 rrm3Δ::KanMX trp1-1 ura3-1*	This study
WRBb-9B	*MAT*<u>**a**</u> *ade2-1 bar1Δ can1-100 his3-11,15 leu2-3,112 RRM3::FLAG::KanMX ura3::URA3/GPD-Tk(7x)*	I. Felipe-Abrio
T1RBb-39A	WRBb-9B *thp1Δ::KanMX*	This study
S3RBb-5B	WRBb-9B *sac3Δ::KanMX*	This study

Table S3 - Primers used in this work

Name	Sequence
GCN4 S	5’-CAGCCAAGTTTATTTGCTTTAAATCC-3’
GCN4 AS	5’-GATACATTTTCGTTGGTTGATTTAGAAC-3’
FAS2 S	5’-ACGCTCCAGCCGTTGAACT-3’
FAS2 AS	5’-CTTCACATCGGTAACACCAGCTT-3’
CIS3 S	5’-AAGCCGGCGCCATCTAC-3’
CIS3 AS	5’-CCAAAGCCAAGTAACCATCTTCA-3’
PMA1 S	5’-ATCGCTATTTTCGCTGATGTTG-3’
PMA1 AS	5’-CGGGCTTTGGAGAGTAAGGA-3’
9716-9863 ChrV S	5’-TGTTCCTTTAAGAGGTGATGGTGAT-3’
9716-9863 ChrV AS	5’-GTGCGCAGTACTTGTGAAAACC-3’
RFL2 S	5’-CCCGCAAATGGGTAATCAAA-3’
RFL2 AS	5’-TTTGCATTGGCTCGAAGATTC-3’
CLN2 S	5’-GAGTCTAACAAGGAAAACCAAAATCC-3’
CLN2 AS	5’-GGGTGGGCCCCCATT-3’
PLB2 S	5’-ACTTTAAACTCAAATTCTTCATCCTCTTC-3’
PLB2 AS	5’-TTCGCTTTGTTTGCCGTAGA-3’
HMS2 S	5’-CAGCATTGCTCCCTCCAACT-3’
HMS2 AS	5’-TGAACCCCAACTGCAAAAAAT-3’
ARG1 S	5’-ACGTCATCATTCTGGGCAGAT-3’
ARG1 AS	5’-CATAGAGGATTCTGTCGGATCGT-3’
SCR1 S	5’-AGGCTGTAATGGCTTTCTGG-3’
SCR1 AS	5’-GTTCAGGACACACTCCATCC-3’
